# An Overview of Reviews on Interprofessional Collaboration in Primary Care: Effectiveness

**DOI:** 10.5334/ijic.5588

**Published:** 2021-06-22

**Authors:** Tania Carron, Cloe Rawlinson, Chantal Arditi, Christine Cohidon, Quan Nha Hong, Pierre Pluye, Ingrid Gilles, Isabelle Peytremann-Bridevaux

**Affiliations:** 1 Center for Primary Care and Public Health (Unisanté), Route de Corniche 10, 1010 Lausanne, Switzerland; 2 Department of Family Medicine, McGill University, 5858, Chemin de la Côte-des-Neiges, Montreal, Quebec, Canada

**Keywords:** interprofessional, collaboration, primary care, effectiveness, overview, review

## Abstract

**Introduction:**

Interprofessional collaboration (IPC) is increasingly used but diversely implemented in primary care. We aimed to assess the effectiveness of IPC in primary care settings.

**Methods:**

An overview (review of systematic reviews) was carried out. We searched nine databases and employed a double selection and data extraction method. Patient-related outcomes were categorized, and results coded as improvement (+), worsening (–), mixed results (?) or no change (0).

**Results:**

34 reviews were included. Six types of IPC were identified: IPC in primary care (large scope) (n = 8), physician-nurse in primary care (n = 1), primary care physician (PCP)-specialty care provider (n = 5), PCP-pharmacist (n = 3), PCP-mental healthcare provider (n = 15), and intersectoral collaboration (n = 2). In general, IPC in primary care was beneficial for patients with variation between types of IPC. Whereas reviews about IPC in primary care (large scope) showed better processes of care and higher patient satisfaction, other types of IPC reported mixed results for clinical outcomes, healthcare use and patient-reported outcomes. Also, reviews focusing on interventions based on pre-existing and well-defined models, such as collaborative care, overall reported more benefits. However, heterogeneity between the included primary studies hindered comparison and often led to the report of mixed results. Finally, professional- and organizational-related outcomes were under-reported, and cost-related outcomes showed some promising results for IPC based on pre-existing models; results were lacking for other types.

**Conclusions:**

This overview suggests that interprofessional collaboration can be effective in primary care. Better understanding of the characteristics of IPC processes, their implementation, and the identification of effective elements, merits further attention.

## Introduction

Due to the growing burden of chronic diseases and aging populations, primary care is facing an increasing number of patients with complex needs, requiring comprehensive, continuous and coordinated care with a variety of healthcare professionals. In response to this burden, new models of care, including interprofessional collaboration (IPC), have been recommended in primary care [1]. The World Health Organization defines IPC as occurring when “two or more individuals from different backgrounds with complementary skills interact to create a shared understanding that none had previously possessed or could have come to on their own” [2]. By enhancing communication, defining a common goal and sharing of expertise between professionals [3], IPC is expected to positively impact care coordination and continuity and finally, patient outcomes [4,5,6,7]. However, IPC is complex and requires that professionals adopt new ways of working “in a manner that effectively utilizes the provider resources to deliver comprehensive primary healthcare in a cost-efficient manner” [3]. Some studies have shown benefits of IPC on patient care across settings (hospital, outpatient, community) [8,9,10], whereas others suggest limited or insufficient evidence [11,12,13] to draw solid conclusions. As the interest in IPC is growing in primary care, an understanding of its effectiveness, implementation processes and mechanisms is necessary.

We conducted an overview (i.e., a review of systematic reviews) to analyze and synthesize results of systematic reviews relating to IPC in the primary care setting, in terms of effectiveness (patient, healthcare professional and cost outcomes), barriers and facilitators of IPC, and theoretical models or conceptual frameworks. In this paper, we present the results on the effectiveness of IPC in primary care.

## Methods

Overviews aim to integrate information from multiple systematic reviews to offer a comprehensive synthesis regarding a specific topic and consider a broader scope than individual systematic reviews [14,15,16]. This type of literature synthesis has been used to manage the amount of information published in systematic reviews, and is referred to by different terminologies such as umbrella review, review of reviews, and overview of reviews [17]. This overview was performed in accordance with the Preferred Reporting Items for Systematic Reviews and Meta-Analysis (PRISMA) statement [18] and recommendations outlined by the Joanna Briggs Institute [17]. The protocol was registered on PROSPERO (CRD42017069922).

### Eligibility criteria

Predefined eligibility criteria concerned three domains. First, reviews had to focus on IPC, which we defined as an ongoing partnership and/or interaction between at least two healthcare professionals from different backgrounds working together to improve patients’ care, according to the WHO definition [2]. More specifically, two forms of collaboration were considered: 1) collaboration within primary care practices/institutions and 2) collaboration between primary care provider(s) (primary care physician(s) (PCP) or primary care nurse(s), such as for example family physicians/practitioners, general physicians/practitioners, nurse practitioners, practice nurses) and healthcare professional(s) working outside the primary care setting. Reviews focusing on interprofessional education, on instruments measuring IPC, or focusing on a specific aspect of IPC were excluded; reviews targeting primarily structural collaboration and not involving interactions between healthcare providers were also excluded. Second, reviews had to explicitly target the primary care setting, as defined by Starfield [19], the Institute of Medicine [1] and the World Health Organization [2]. When the setting was not clearly specified, the IPC process had to include at least a primary care provider. Third, reviews were eligible irrespective of the type of primary studies they considered (quantitative studies with or without meta-analysis, qualitative studies or a combination of qualitative, quantitative and/or mixed methods studies); reviews targeting conceptual frameworks (including typologies and taxonomies) were also eligible. Finally, reviews had to be conducted systematically [20]: rigorous and explicit methodology in terms of search strategy, eligibility criteria, data extraction, quality appraisal, and synthesis of results.

### Search strategy

The search strategy was elaborated with a librarian; MeSH terms and words relating to the concepts of IPC, primary care and review, were included (S1 Table). The search was carried out on May 10^th^, 2017 in nine databases: MEDLINE, EMBASE, CINAHL, PsycINFO, Cochrane Database of Systematic Reviews, Database of Abstract Reviews of Effects (DARE), JBI Database of Systematic Reviews and Implementation Reports, PROSPERO, and Epistemonikos. An update was performed on January 31^st^, 2019. Reference lists of included reviews were checked for additional reviews.

### Study selection and data extraction

We used the Covidence platform to carry out a two-stage screening process: first, titles and abstracts and then, full-text papers. Data from eligible reviews were extracted using a standardized predefined data extraction form (S2 Table), and corresponding authors were contacted for missing or incomplete data. Three authors (T.C., C.R. and C.A.) took part in these processes, so that for all articles two independent reviewers assessed eligibility criteria at both stages and extracted data. Disagreements were resolved during discussions between authors. When necessary, a fourth author was consulted for final decision (I.P.B.).

### Quality assessment

Two independent reviewers (T.C., C.R. or C.A.) used the ROBIS tool [21] to assess the methodological quality of the included reviews. This tool contains 24 questions organized in four domains (study eligibility criteria, identification and selection of studies, data collection and study appraisal, and synthesis and findings). A *Low, High* or *Unclear* risk of bias (RoB) was attributed to each domain and to the review in general. No reviews were eliminated according to RoB; results served as an indicative purpose to inform on the quality of the results.

### Degree of overlap

To address the representation of primary studies in more than one review and avoid interpretation biases [22], the degree of overlap was calculated using the Corrected Covered Area (CCA) measure [23]. A CCA value ≤ 5% was considered as a slight overlap, whereas values ≥ 15% as a very high overlap [23].

### Synthesis of results

Reviews were firstly categorized according to the type of IPC targeted by the review, in terms of setting and type of healthcare professionals involved, as defined by the authors of the review. Then, since the methods used to synthesize results in the included reviews were very diverse, ranging from narrative synthesis to meta-analysis, an overall quantitative synthesis was not possible. Instead, we synthesized the results by aggregating them according to six broad categories of patient outcomes: clinical outcomes, medication outcomes, healthcare use, processes of care, quality of life (QoL), functioning, other patient-reported outcome measures, and patient satisfaction (***Table 1***). Results were coded as follows: (1) improvement (+), when a review reported improvements in all outcomes from a category of outcomes (e.g. for meta-analysis, a standardized mean difference; for narrative synthesis, a statistical significant difference in primary studies before/after the intervention or versus a control group); (2) worsening (–), corresponding to a review reporting worsening in all outcomes from a category; (3) mixed results (?), when a review reported mixed findings (e.g. improvement of one outcome but no change or worsening effect in another) between primary studies reporting the same outcome or between different outcomes of a given category; (4) no change (0) for a review reporting no change in the outcomes from a category. Codes were attributed independently by two reviewers (T.C., C.R.), and disagreements were resolved during discussion. Results are presented narratively and in the form of a table. When a meta-analysis had been carried out in a review, it was specified in the table (marked with a “√”). Professional, organizational and cost-related outcomes were narratively reported.

**Table 1:** Categorization of patient outcomes^a^.


CATEGORIES OF PATIENT OUTCOMES	EXAMPLES OF INCLUDED OUTCOMES

Clinical outcomes	Depression/anxiety scale, HbA1c level, blood pressure, lipids, other clinical outcomes, symptomatology, recovery, remission, mortality, morbidity, survival

Medication outcomes	Medication use, medication adherence, compliance with medication, number of prescribed drugs

Healthcare use	Hospital admissions, hospital utilization, medical service use, emergency department visits, length of stay, usage, readmission rate, time to readmission

Processes of care	Provision of recommended tests and preventive services, adherence to recommended care guidelines (vaccination, monitoring), improved accessibility, reduced waiting times, treatment adequacy, appropriate medications

QoL, functioning, other PROMs	Patient quality of life, physical/emotional/and social functioning, patient health behaviors, health practices (lifestyle, self-care), self-perceived health, QALY

Patient satisfaction	Patient satisfaction, attitudes and perceptions of care


^a^ HbA1c: Haemoglobin bA1c; PROMs: Patient-Reported Outcome Measures; QALY: Quality-Adjusted Life Years; QoL: Quality of Life.

## Results

### Search results

**Figure 1:**
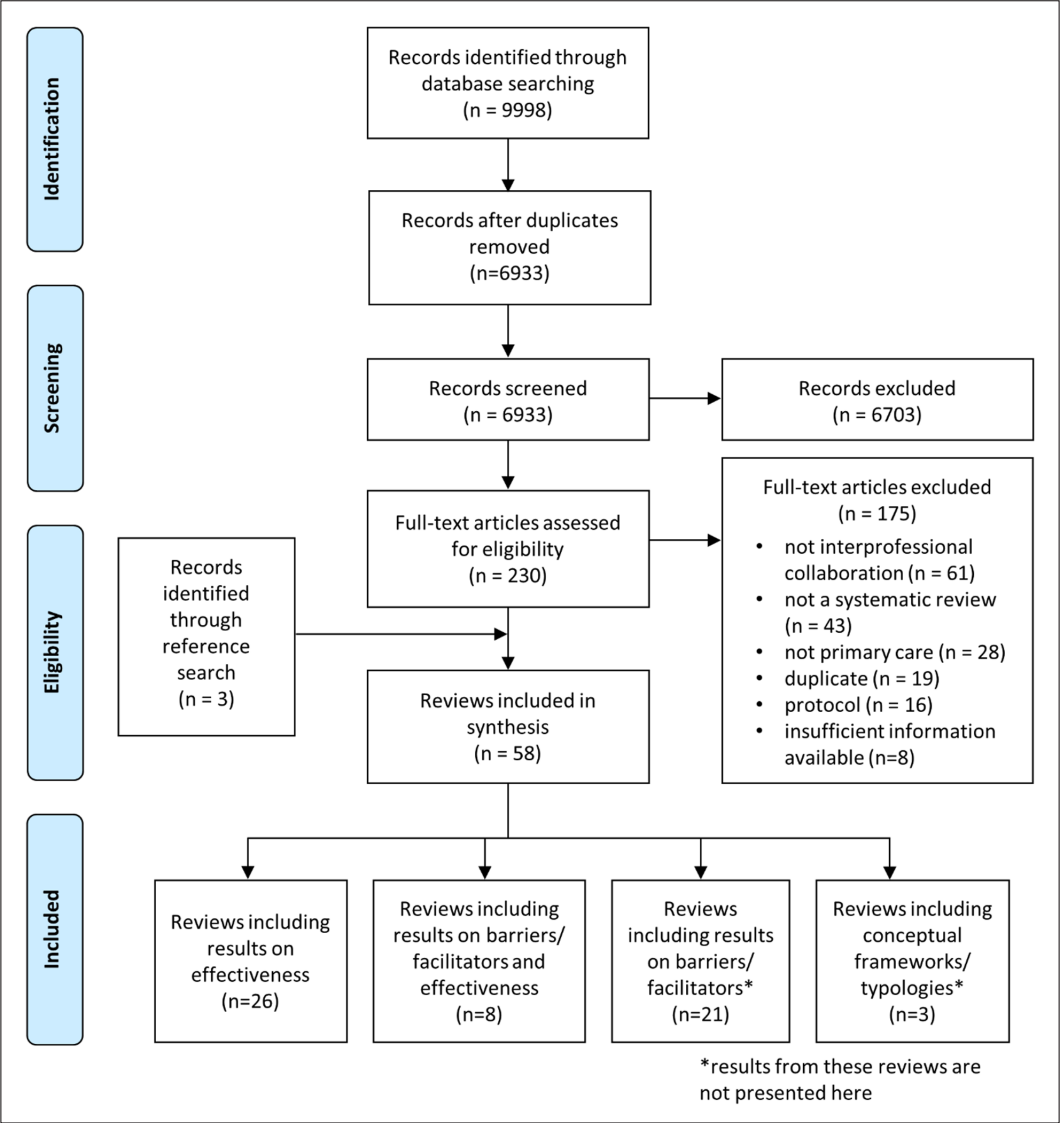
Flow chart.

Of the 9998 identified records, 230 full-text articles were assessed, and 58 reviews met the eligibility criteria. Of these, 34 were related to IPC effectiveness and were included in this paper (***Figure 1***). The 34 identified reviews included between 5 and 206 primary studies, covering a total of 1,177 publications. The CCA was 1.3 %, indicating a slight degree of overlap.

### Characteristics of included reviews

**Figure 2:**
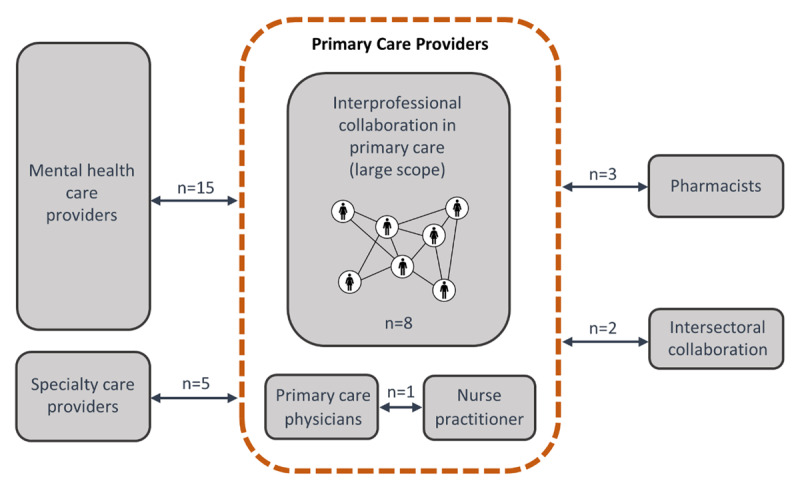
Six types of interprofessional collaboration identified. *Note*: professionals working outside the primary care setting collaborate with at least a primary care physician and can include other primary care providers.

Of the 34 reviews included in this overview, eight were mixed methods reviews (i.e. integrating results of qualitative, quantitative and/or mixed methods studies) and 26 were quantitative reviews (of which 12 included a meta-analysis). Based on the scope of the reviews (in terms of setting and type of healthcare professionals involved), six types of IPC were defined as follows: (1) *IPC in primary care (large scope)* for reviews evaluating the effects of interprofessional primary care teams, without targeting specific professionals (in these reviews studies included two or more different professionals in the collaboration process, such as PCPs, primary care nurses, specialist physicians and allied health professionals, working within or outside the practice) (n = 8); (2) *PCP-nurse practitioner collaboration* corresponded to reviews focusing on collaboration between physicians and nurses in primary care, for example by assessing the effects of PCPs-nurse practitioners’ co-management of primary care patients (n = 1); (3) *PCP-specialty care provider collaboration* included reviews targeting collaboration between a PCP and a specialist (e.g. palliative care providers, oncologists, psychiatrists, cardiologists, diabetes specialist nurses) and investigated the effects of the implementation of various interventions, including face-to-face meetings/case conferences, telephone discussions, shared care records and referral guidelines (n = 5); (4) *PCP-pharmacist collaboration* corresponded to reviews specifically addressing collaboration between PCP and pharmacists, such as evaluating medication review interventions or PCP-pharmacist co-location (n = 3); (5) *PCP-mental health care provider collaboration* contained reviews devoted to primary mental health interventions, such as “Collaborative care” models (n = 15). The latter generally included four main components: a multi-professional approach to patient care (e.g. PCP, mental health specialist, and case manager), a structured management plan, scheduled patient follow-ups, and enhanced inter-professional communication (team meetings, shared medical records, etc.). The final type of IPC was (6) *intersectoral collaboration*, and included reviews on the collaboration between different sectors (primary care providers and care home staff, primary care and public health) (n = 2). The 34 reviews were assigned to the corresponding category according to their scope (***Figure 2***). Risk of bias was rated as low, high and unclear in 14, 16 and 4 reviews, respectively. Characteristics of the reviews are presented in S3 Table.

### Patient outcomes

Most reviews reported clinical outcomes (n = 31); QoL, functioning and patient-reported outcome measures (PROMs) were reported in 20 reviews, medication outcomes in 14, processes of care in 12, patient satisfaction in 12, and healthcare use in 11 reviews. In addition, 18 reviews reported intervention characteristics that were associated with effectiveness. After presenting a summary of the main findings for each type of IPC in ***Table 2*** (see S4 Table for detailed results), we describe the synthesized results narratively, by type of IPC.

**Table 2:** Results from included reviews for patient outcomes (n = 34).


TYPE OF COLLABORATION	AUTHORS, YEAR	META-ANALYSIS	CLINICAL OUTCOMES	MEDICATION OUTCOMES	HEALTHCARE USE	PROCESS OF CARE	QOL, FUNCTIONING, OTHER PROMS	PATIENT SATISFACTION

**Interprofessional collaboration in primary care (large scope)**	Barrett, 2007 [24]		+			+	+	+

	DeLoach, 2018 [25]		+					

	Gougeon, 2017 [26]		?		?		+	+

	Martin, 2010 [27]		?		?		?	+

	Proia, 2014 [28]		+	+			+	+

	Schepman, 2015 [29]		?		?		?	

	Trivedi, 2013 [30]		?		?	+	?	+

	Wranik, 2019 [31]		?	?	+	+	?	

**PCP – nurse practitioner collaboration**	Norful, 2017 [32]		?			+	?	

**PCP – specialty care provider collaboration**	Carmont, 2017 [36]				+		?	

	Foy, 2010 [37]	√	+					

	Mitchell, 2002 [33]		?		?	+	?	+

	Mitchell, 2015 [34]		?	?	?	?	?	?

	Smith, 2017 [35]	√	?	+	?	?	?	?

**PCP – pharmacist collaboration**	Hazen, 2017 [39]		0	0			0	

	Kwint, 2013 [40]			?	0	?	0	

	Health Quality Ontario, 2009 [38]	√	+					

**PCP – mental healthcare provider collaboration**	Archer, 2012 [41]	√	+	+			+	+

	Bower, 2006 [45]	√	+	+				

	Butler, 2008 [52]		+					

	Coventry, 2014 [46]	√	+	+				

	Craven, 2006 [54]		?	?	?	?	?	?

	Dham, 2017 [55]		?	+		+	?	?

	Fuller, 2011 a [53]		+			+		

	Gilbody, 2006 [47]	√	+					

	Gunn, 2006 [48]		+					

	Huang, 2013 [50]	√	+	+				

	Muntingh, 2016 [51]	√	+					

	Panagioti, 2016 [49]	√	+					

	Sighinolfi, 2014 [43]	√	+					

	Thota, 2012 [42]	√	+	+			+	+

	van Steenbergen-Weijenburg, 2010 [44]		+				+	

**Intersectoral collaboration (primary care with nursing home, public health)**	Davies, 2011 [56]		?	?				

	Martin-Misener, 2012 [57]					+	+	


+ Improvement: a review reports improvements in all outcomes from a category of outcomes (e.g. standardized mean difference, statistical significant difference before/after the intervention or versus a control group). ? Mixed results: a review reports mixed findings (e.g. improvement of one outcome but no change or worsening effect in another) between primary studies reporting the same outcome or between different outcomes of a given category. 0 No change: a review reports no change in the outcomes from a category of outcomes. √ The review included a meta-analysis.

#### IPC in primary care (large scope) (n = 8)

Among the eight reviews [24,25,26,27,28,29,30,31] on IPC in primary care, five [26,27,29,30,31] reported mixed results on clinical outcomes, while three [24,25,28] reported an improvement: reduction of HbA1c, mean systolic blood pressure (SBP) and diastolic blood pressure (DBP) levels, and reduction of body mass index in diabetic patients receiving care from an interprofessional team (i.e. when a nurse or a pharmacist collaborated with the PCP) [24,25], and a reduction in SBP and DBP in patients with primary hypertension receiving team-based care when compared to usual care [28]. Improvement in QoL, functioning or other PROMs (such as improved self-care, lifestyle, decreased functional decline) were reported in three reviews [24,26,28], whereas four reviews reported mixed results for this category of outcome [27,29,30,31]. Whereas the addition of practice nurses to primary care teams expanded the range of services provided, the addition of a pharmacist led to conflicting evidence on medication use in patients with chronic conditions [31]. One review [31] showed a decrease in visits to the emergency room and four reported mixed results on healthcare use [26,27,29,30]. Positive effects on care processes (e.g. access, provision of recommended tests) and patient satisfaction were reported in three [24,30,31] and five [24,26,27,28,30] reviews, respectively.

Regarding the relationship between the type of interventions delivered and their effectiveness, two reviews found that models considering individual care plans tended to report a greater number of favorable outcomes and greater effect sizes, compared to other collaborative models [26,29]. Moreover, two reviews evaluating the effectiveness of IPC among elderly patients specifically [26,30], and one review comparing the effectiveness of IPC according to the target population of patients [29], found that, despite some positive effects observed on some clinical outcomes, patient-reported measures and processes of care, evidence remained weak for interventions delivered to elderly people. Finally, a review addressing team-based care for patients with hypertension observed a larger improvement in blood pressure outcomes if the additional team member (pharmacist or nurse) was able to make or propose changes to medications, compared to adherence support and information on medication and hypertension only [28].

#### PCP-nurse practitioner collaboration (n = 1)

In the only review included for this type of IPC, which compared the effect of PCP-nurse practitioner co-management to individual physician-led care for primary care patients, significantly more recommended care guidelines (e.g. discussion of medication side effects, diabetic control monitoring, vaccinations for patients with chronic diseases, examinations) were completed when PCP-nurse practitioner co-management was present. However, mixed findings were reported for clinical outcomes and no significant differences were observed in patients’ quality of life [32].

#### PCP-specialty care provider collaboration (n = 5)

These types of reviews focused on models integrating primary and secondary care providers [33] in chronic care [34,35], palliative care [36], and psychiatry and endocrinology care [37]. One review evaluating the effects of interactive communication (timely and two-way exchange of pertinent clinical information) between PCPs and specialists in psychiatry and endocrinology care showed significant improvement in clinical outcomes for depression and diabetes (HbA1c levels), especially when interventions aiming at improving the quality of information exchange (such as structured forms, pathways, or reports) were included [37]. In one review that compared shared/integrated care to usual care for patients with chronic conditions [35], medication appropriateness and adherence for depression, as well as response to depression treatment and recovery from depression, were improved. Effects on mean depression scores were modest and evidence was lacking for other chronic conditions. Another review targeting shared/integrated care delivered at the primary-secondary interface for complex and chronic diseases reported mixed results for all categories of outcomes: some studies showed improvements in clinical outcomes and healthcare utilization (lower hospital admission rates and length of stay) while others reported no change [34]. Regarding healthcare use, only one review on PCP engagement in palliative care showed positive outcomes for hospital use (reduced readmissions, shortened length of stay) [36]. GP involvement with specialists led to consistently greater patient satisfaction and better processes of care in one review [33], while two others showed mixed results [34,35]. All reviews reported mixed results for PROMs.

#### PCP-pharmacist collaboration (n = 3)

This type of collaboration included three reviews. One review on multidisciplinary community care for patients with type 2 diabetes involving at least a pharmacist and a PCP [38] reported improvement in patient outcomes, with a statistically and clinically significant reduction in HbA1c and systolic blood pressure compared to usual care. Another review that examined the (partly) co-location of a non-dispensing clinical pharmacist with a primary care team to improve medication use found no association between the degree of integration and improvement in health outcomes, except when results were stratified according to the type of pharmacy services provided: a positive association was found for patient-centered services (e.g. polypharmacy), while a negative association was observed for disease-specific services (e.g. diabetes, chronic obstructive pulmonary disease) [39]. The third review assessed medication review interventions involving pharmacists and GPs for home-dwelling patients (≥70); it showed a significant association between the intensity of GP-pharmacist collaboration in medication review and the implementation rate of recommendations following drug-related problems [40].

#### PCP-mental health care provider collaboration (n = 15)

All 15 reviews included in this type of IPC focused on collaborative care (i.e. a multi-professional intervention involving a GP, a mental health specialist and a case manager) or other collaborative models in primary mental health care. Whereas 13 out of 15 reviews reported significant improvements in clinical outcomes for depression and anxiety (***Table 2***) [41,42,43,44,45,46,47,48,49,50,51,52,53], two focusing on mental health in general [54] and psychotic disorders in older patients [55] reported mixed results for clinical outcomes. Interventions including a recognized psychological treatment model [46], a planned supervision from the case manager [45,47], or systematic patient identification [45,49], were significantly more effective for depressive symptoms. A review examining the connection between PCPs and mental health providers/services found that studies with positive clinical outcomes included most of the time care management, enhanced communication, consultation liaison (i.e. explicit arrangement to provide expert level advice to PCP) and local protocols [53]. Additionally, interventions with higher fidelity to Gunn’s definition of collaborative care [48] showed higher efficacy [43], as did interventions implemented by community-based organizations including nurses as case managers [42]. Also, the integration of mental health services in primary care settings showed improvements for patients with mental health disorder or alcohol related substance abuse on symptom severity, treatment response, and remission when compared to usual care [52]. Regarding medication outcomes, collaborative care showed improvements for medication use [41,45,46], and adherence to treatment [42,50,55]. Finally, among the few reviews reporting patient satisfaction and PROMs, about half reported improved outcomes.

#### Intersectoral collaboration (n = 2)

Two reviews were included in this type of collaboration [56,57]. In the review focusing on the integration between healthcare professionals and nursing home staff, the important heterogeneity of outcomes and interventions made the comparison challenging; although some improvements were observed, the majority of included studies showed that the intervention had mixed or no effect on clinical and medication outcomes in comparison to the control group [56]. The review focusing on collaboration between primary care and public health [57] reported improved processes of care and QoL, functioning and other PROMs.

### Healthcare professional (n = 6), organizational (n = 2) and cost outcomes (n = 11)

Professional and organizational outcomes were under-reported in comparison to patient outcomes. Six reviews addressed healthcare professional outcomes, reporting increased healthcare professional satisfaction [24,31] and more positive experiences and perceptions of IPC (such as improved communication, better understanding of responsibility and roles) [24,36,57]. However, an increase in the ratio of non-clinical to clinical staff deteriorated team climate in one review [31]. Professionals’ development of new knowledge and skills [24] and improved GP clinical behaviors [33,34] were also reported.

Organizational outcomes were reported in two reviews [24,57]. The first one reported better use of resources and access to services, shorter waiting times and more comprehensive care from IPC models, in comparison to a uni-professional model for delivering care [24]. The second review reported that intersectoral collaboration between primary care and public health improved access to care, efficiency (such as timelier reporting) and strengthened the delivery of care processes [57].

With regard to cost outcomes, all 11 reviews reported mixed results and/or insufficient data to conclude on the cost or cost-effectiveness of IPC models in primary care [24,26,30,33,34,35,44,52,53,55,57]. Indeed, most reviews reported that economic data were limited and that primary studies used different methods to measure costs and benefits, different timeframes and economic indicators, making comparison impossible. Despite this heterogeneity between primary studies, reviews targeting collaborative care [44,52,53,54,55] or shared care [35] tended to report more positive results on cost outcomes, especially if other outcomes such as quality-adjusted life years (QALYs) and/or depression-free days were considered when measuring cost benefits [55]. Moreover, in a review evaluating the cost-effectiveness of collaborative care for the treatment of major depressive disorder in primary care, authors concluded that even though collaborative care is effective (in terms of QALYs and depression-free days), it remained more expensive than usual care in most cases [44]. Detailed results are presented in S5 Table.

## Discussion

Results of this overview of reviews show that a large body of evidence regarding the effectiveness of IPC in primary care setting exists. Across the six different types of IPC identified, clinical, medication and process of care outcomes, as well as patient satisfaction, improved in the majority of the included reviews. This service-oriented improvement pattern seems promising and quite robust, in the sense that it appears to be generalized across populations, primary care settings and IPC types. In contrast, the impact of IPC on decreasing healthcare use, such as hospital admission rates, or on improving QoL, functioning and other PROMs, remains unclear as most reviews showed mixed results. Worsening of patient-related outcomes was not reported for any category of outcomes.

Our results suggest that evidence is in fact lacking for outcomes relating to healthcare use, QoL, functioning and other PROMs. There are several explanations for this. First, effects of IPC on clinical, medication and process of care outcomes are probably easier to highlight than effects on healthcare use or quality of life because the latter involve multiple factors such as patients’ socio-economic status or education level [58]. Therefore, the latter cannot be considered as direct outcomes of IPC. Second, healthcare use, QoL, functioning and other PROMs were often not included as main outcomes in the studies. In fact, many reviews included in our overview did not provide data on these types of outcomes. Whereas clinical outcomes were reported more often, they do not appear as the most pertinent when it comes to evaluating interprofessional collaboration. In fact, a recent study [59] that interviewed 283 primary healthcare providers from 14 different health professions working in interprofessional primary healthcare teams in the province of Ontario, Canada, concluded that the most appropriate indicator for evaluating the performance of IPC was patient experience, then came patient health status, followed by intra-agency referrals, workload measurements and staff experiences. Indeed, besides the question of which patient-related outcomes to measure, our overview also revealed that very few systematic reviews evaluated the impact of IPC on healthcare professional and organizational outcomes. From our results, IPC in primary care setting appears to be beneficial for professional, organizational and cost-related outcomes, but evidence is limited. In the literature, professional- and organizational-related outcomes remain in fact under-considered [60,61], even though professionals’ satisfaction and patients’ outcomes are known to be closely associated [62]. Indeed, growing GP dissatisfaction in primary care from organizational factors (workload, pressure) [63] were reported. This is coherent with our findings regarding barriers and facilitators to IPC, which were mostly identified at the organizational and inter-individual (between professionals) levels (see the other paper from Carron et al. in the same issue), suggesting these to be a basis for successful collaboration [64,65,66].

Another important result is that IPC effectiveness seems to vary not only across indicators, but also between types of collaboration. This highlights the fact that not all types of IPC interventions are effective. If we look at the variations in the way IPC was implemented across the six types of IPC in terms of intensity of the collaboration, these range from consultation or referral where professionals solely exchange information to interdependent co-provision of care with shared decision-making processes. Although we cannot be very specific about the exact types of interventions that are most effective, our results tend to show that the higher the intensity of the collaboration, the more it improves patient outcomes [26,40,48]. In fact, reviews including interventions based on pre-existing and well-defined models, such as collaborative care models, showed most improvements in outcomes (“+”). This may be partly due to the fact that these reviews, due to the homogeneity in the interventions from the primary studies, were able to conduct meta-analyses and thus demonstrate a positive effect. In contrast, reviews which only provided a narrative synthesis due to methodological heterogeneity between primary studies, often reported mixed results. Another explanation is found in the characteristics of the models implemented. Unlike the other types of IPC evaluated, collaborative care models included scheduled pro-active patient follow-ups, a component that is not related to the IPC phenomenon as such and may have contributed to the effectiveness of these interventions. However, as is the case with the evaluation of complex interventions that consider a variety of single elements interacting between each other, it is not possible to be sure that this specific component was particularly effective. Some reviews that analyzed the association between intervention characteristics and patient outcomes were able to highlight some “active ingredients” of IPC, such as the use of individual care plans [26,29]. Other reviews found collaboration to be effective only for certain populations (e.g. non-specific population rather than elderly people [29]) or certain types of services delivered (e.g. patient-centered rather than disease-specific pharmacy services [39]).

To our knowledge, this is the first overview of reviews that targeted the effectiveness of IPC in primary care and aimed at providing a broad perspective on the topic. Despite the use of rigorous and state-of-the-art methodology, some limitations need to be considered while interpreting the results. The first limitation relates to our search strategy. Despite our efforts to translate our two main concepts (IPC and primary care) in a comprehensive search strategy and in the operationalization of our eligibility criteria, it is possible that some reviews might not have been selected due to the lack of consensual definition of these concepts. Also, we found relatively few reviews focusing on more specific IPC such as collaboration between PCPs and nurses, pharmacists, and specialty care providers. This may be partly explained by the fact that our search strategy did not include words related to the type of healthcare professionals involved in IPC, as our goal was to be as broad as possible in the inclusion of reviews. Second, even though our search included nine recognized databases and was carried out using systematic methods, we did not search for grey literature. Third, as an overview is based on the review authors’ interpretation and reporting of primary studies’ results, differences in the levels of information provided, combined with the heterogeneity of interventions, designs and contexts, made the homogenization and synthesizing of results challenging. For this reason, we decided not to compare reviews and present trends in patient-related outcomes for each review separately. Fourth, we had to deal with a common challenge when carrying out an overview of reviews: the overlap and scope mismatch [22]. Despite the fact that we found only a slight overlap between the 34 reviews, some primary studies were included in more than one review, which could have led to the over-representation of some study results. In addition, the six types of IPC we identified were not mutually exclusive due to some overlap between the scopes of the included reviews. Finally, the last limitation relates to the overall quality of the systematic reviews included, which was moderate. However, this should not preclude the presentation of overall trends of results.

## Conclusion

Our overview suggests that, overall, interprofessional collaboration in the primary care setting can bring benefits to patients. However, interventions involving IPC are complex and diverse, which suggests that the evaluation of IPC effectiveness in primary care should take into account particularities relating to the intervention. Future research needs to recognize this diversity and attempt to identify key characteristics of IPC which work in specific contexts, as well as the common components that could contribute to the success of IPC throughout the different forms, contexts and populations in terms of outcomes that matter to patients. Given the complexity of IPC, a better understanding of the characteristics of IPC processes in the different practice and organizational contexts, along with the identification of the most effective IPC elements and their relationships, merits further attention. In particular, the use of realist evaluation, which provides an in-depth understanding of what works, for whom, and in what circumstances, could be particularly appropriate in this context. Cost-, organizational- and professional-related outcomes also require further investigation.

## Additional File

The additional file for this article can be found as follows:

10.5334/ijic.5588.s1 Supplementary material. Tables S1–S5.
