# Supplementary material for: An Overview of Reviews on Interprofessional Collaboration in Primary Care: Effectiveness
Source: Int J Integr Care. 2021 Jun 22;21(2):31. doi: 10.5334/ijic.5588 (PMC8231476; doi:10.5334/ijic.5588)
Supplement: Supplementary material. — Tables S1–S5. [file ijic-21-2-5588-s1.pdf]

Table S1: search strategy details

**Medline Ovid - Ovid MEDLINE(R) and Epub Ahead of Print, In-Process & Other Non-Indexed Citations and Daily**	**(1)** (Cooperative Behavior/ or Interprofessional Relations/ or Physician-Nurse Relations/ or Interdisciplinary Communication/ or Patient Care Team/ or (collaborati* or cooperati* or co-operati* or crossdisciplinar* or cross-disciplinar* or integrated care or interdisciplin* or inter-disciplin* or interprofession* or inter-profession* or multidisciplin* or multi-disciplin* or multiprofession* or multi-profession* or team* or transdisciplin* or trans-disciplin*).ti,ab,kf.)<br>AND<br>**(2)** (Community Health Centers/ or Community Health Services/ or Community Health Nursing/ or Community Mental Health Services/ or Community Pharmacy Services/ or Home Care Services/ or General Practice/ or Family Practice/ or General Practitioners/ or Physicians, Family/ or Physicians, Primary Care/ or Primary Health Care/ or Office Visits/ or Primary Care Nursing/ or (((community or primary) adj3 (care or health or healthcare or practitioner*)) or (community based medicine or (communit$3 adj5 nurse?) or community nursing or community pharmac* or family doctor* or family medicine or family physician* or family medical practice* or family practi* or GP or GPs or general medical practice or general medicine or general physician* or general practi* or health center? or health centre? or medical home* or primary practice)).ti,ab,kf.<br>AND<br>**(3)** (meta-analysis/ or meta-analysis as topic/ or (meta analy* or metanaly* or metaanaly* or meta regression).ti,ab. or ((systematic* or evidence*) adj3 (review* or overview*)).ti,ab. or (search strategy or search criteria or systematic search or study selection or data extraction).ab. or (search* adj4 literature).ab. or (concept synthesis or conceptual review or critical interpretive synthesis or framework synthesis or integrative review or integrative literature review or literature review or meta-data-analysis or meta-ethnography or (meta adj2 narrative) or meta-study or meta-synthesis or mixed method* review or mixed research synthesis or mixed studies review or narrative review or narrative synthesis or realist review or realist synthesis or scoping review or scoping study or qualitative evidence synthesis or qualitative interpretive meta-synthesis or qualitative research synthesis or qualitative systematic review or thematic synthesis or theoretical synthesis).ti,ab.)
**PubMed**	**(1)** ((collaborati*[tiab] or cooperati*[tiab] or co-operati*[tiab] or crossdisciplinar*[tiab] or cross-disciplinar*[tiab] or integrated care[tiab] or interdisciplin*[tiab] or inter-disciplin*[tiab] or interprofession*[tiab] or inter-profession*[tiab] or multidisciplin*[tiab] or multi-disciplin*[tiab] or multiprofession*[tiab] or multi-profession*[tiab] or team*[tiab] or transdisciplin*[tiab] or trans-disciplin*[tiab])

	AND<br>**(2)** (community based medicine[tiab] or community care[tiab] or community health[tiab] or community healthcare[tiab] or community mental health service*[tiab] or community mental health nursing[tiab] or community nurse[tiab] or community nurses[tiab] or community nursing[tiab] or community pharmac*[tiab] or community practitioner*[tiab] or family doctor*[tiab] or family medicine[tiab] or family physician*[tiab] or family medical practice*[tiab] or family practi*[tiab] or GP[tiab] or GPs[tiab] or general medical practice[tiab] or general medicine[tiab] or general physician*[tiab] or general practi*[tiab] or health center[tiab] or health centers[tiab] or health centre[tiab] or health centres[tiab] or medical home*[tiab] or primary care[tiab] or primary health[tiab] or primary healthcare[tiab] or primary practice[tiab] or primary practitioner*[tiab])<br>AND<br>**(3)** (meta analy*[tiab] or metanaly*[tiab] or metaanaly*[tiab] or meta regression[tiab] or evidence overview*[tiab] or evidence review*[tiab] or systematic overview*[tiab] or systematic review*[tiab] or search strategy[tiab] or search criteria[tiab] or systematic search[tiab] or study selection[tiab] or data extraction[tiab] or literature search*[tiab] or search literature[tiab] or concept synthesis[tiab] or conceptual review[tiab] or critical interpretive synthesis[tiab] or framework synthesis[tiab] or integrative review[tiab] or integrative literature review[tiab] or literature review[tiab] or meta-data-analysis[tiab] or meta-ethnography[tiab] or meta-narrative[tiab] or meta-study[tiab] or meta-synthesis[tiab] or mixed method review[tiab] or mixed methodological review[tiab] or mixed methodology review or mixed methods review[tiab] or mixed research synthesis[tiab] or mixed studies review[tiab] or narrative review[tiab] or narrative synthesis[tiab] or realist review[tiab] or realist synthesis[tiab] or scoping review[tiab] or scoping study[tiab] or qualitative evidence synthesis[tiab] or qualitative interpretive meta-synthesis[tiab] or qualitative research synthesis[tiab] or qualitative systematic review[tiab] or thematic synthesis[tiab] or theoretical synthesis[tiab]))<br>**NOT Medline[sb]**
**Embase (Embase Ovid on May 10th 2018, Embase.com on January 31st 2019)**	**(1)** ('Cooperation'/de OR 'Public Relations'/de OR 'Teamwork'/de OR 'Doctor Nurse Relation'/de OR 'Interdisciplinary Communication'/de OR (collaborati* or cooperati* or co-operati* or crossdisciplinar* or cross-disciplinar* or integrated care or interdisciplin* or inter-disciplin* or interprofession* or inter-profession* or multidisciplin* or multi-disciplin* or multiprofession* or multi-profession* or team* or transdisciplin* or trans-disciplin*):ti,ab,kw)<br>AND<br>**(2)** ('Health Center'/de OR 'Community Care’/de OR 'Community Health Nursing’/de OR 'Community Mental Health Center’/de OR 'Home Care’/de OR 'General Practice’/de OR 'General Practitioner’/de OR 'Primary Health Care’/de OR 'Primary Medical Care’/de

	OR (('community' or 'primary') NEAR/3 ('care' or 'health' or 'healthcare' or 'practitioner*')):ti,ab,kw OR 'community based medicine':ti,ab,kw OR (communit* NEAR/5 nurse*):ti,ab,kw OR ('community nursing' or 'community pharmac*' or 'family doctor*' or 'family medicine' or 'family physician*' or 'family medical practice*' or 'family practi*' or 'GP' or 'GPs' or 'general medical practice' or 'general medicine' or 'general physician*' or 'general practi*' or 'health center*' or 'health centre*' or 'medical home*' or 'primary practice'):ti,ab,kw)<br>AND<br>**(3)** ('Meta analysis'/de OR 'Meta analysis (topic)'/de OR 'Systematic review'/de OR 'Systematic review (topic)'/de OR ('meta analy*' or 'metanaly*' or 'metaanaly*' or 'meta regression'):ti,ab OR (('systematic*' or 'evidence*') NEAR/3 ('review*' or 'overview*')):ti,ab OR ('search strategy' or 'search criteria' or 'systematic search' or 'study selection' or 'data extraction'):ab OR ('search*' NEAR/4 'literature'):ab OR ('concept synthesis' or 'conceptual review' or 'critical interpretive synthesis' or 'framework synthesis' or 'integrative review' or 'integrative literature review' or 'literature review' or 'meta-data-analysis' or 'meta-ethnography' or (meta NEAR/2 narrative) or 'meta-study' or 'meta-synthesis' or 'mixed method* review' or 'mixed research synthesis' or 'mixed studies review' or 'narrative review' or 'narrative synthesis' or 'realist review' or 'realist synthesis' or 'scoping review' or 'scoping study' or 'qualitative evidence synthesis' or 'qualitative interpretive meta-synthesis' or 'qualitative research synthesis' or 'qualitative systematic review' or 'thematic synthesis' or 'theoretical synthesis'):ti,ab)
**CINAHL**	**(1)** MH ("Cooperative Behavior" OR "Interprofessional Relations" OR "Nurse-Physician Relations" OR "Multidisciplinary Care Team") OR TI (collaborati* OR cooperati* OR co-operati* OR crossdisciplinar* OR cross-disciplinar* OR integrated care OR interdisciplin* OR inter-disciplin* OR interprofession* OR inter-profession* OR multidisciplin* OR multi-disciplin* OR multiprofession* OR multi-profession* OR team* OR transdisciplin* OR trans-disciplin*) OR AB (collaborati* OR cooperati* OR co-operati* OR crossdisciplinar* OR cross-disciplinar* OR integrated care OR interdisciplin* OR inter-disciplin* OR interprofession* OR inter-profession* OR multidisciplin* OR multi-disciplin* OR multiprofession* OR multi-profession* OR team* OR transdisciplin* OR trans-disciplin*)<br>AND<br>**(2)** MH ("Community Health Centers" OR "Community Health Services" OR "Community Health Nursing" OR "Community Mental Health Services" OR "Home Health Care" OR "Family Practice" OR "Physicians, Family" OR "Primary Health Care" OR "Office Visits") OR TI ((community or primary) N3 (care or health or healthcare or practitioner*)) OR TI ((community based medicine or (communit*3 N5 nurse*2) or community nursing or community pharmac* or family doctor* or family medicine or family physician* or family medical practice* or family practi* or GP or GPs or general medical practice or general medicine or general

	physician* or general practi* or health center*2 or health centre*2 or medical home* or primary practice)) OR AB ((community or primary) N3 (care or health or healthcare or practitioner*)) OR AB ((community based medicine or (communit*3 N5 nurse*2) or community nursing or community pharmac* or family doctor* or family medicine or family physician* or family medical practice* or family practi* or GP or GPs or general medical practice or general medicine or general physician* or general practi* or health center*2 or health centre*2 or medical home* or primary practice))<br>AND<br>**(3)** MH ("Meta Analysis" OR "Systematic Review") OR PT ("Meta Analysis OR "Systematic Review") OR TI (meta analy* or metanaly* or metaanaly* or meta regression) OR TI ((systematic* or evidence*) N3 (review* or overview*)) OR TI ((search strategy or search criteria or systematic search or study selection or data extraction) or (search* N4 literature) or (concept synthesis or conceptual review or critical interpretive synthesis or framework synthesis or integrative review or integrative literature review or literature review or meta-data-analysis or meta-ethnography or (meta N2 narrative) or meta-study or meta-synthesis or mixed method* review or mixed research synthesis or mixed studies review or narrative review or narrative synthesis or realist review or realist synthesis or scoping review or scoping study or qualitative evidence synthesis or qualitative interpretive meta-synthesis or qualitative research synthesis or qualitative systematic review or thematic synthesis or theoretical synthesis)) OR AB (meta analy* or metanaly* or metaanaly* or meta regression) OR AB ((systematic* or evidence*) N3 (review* or overview*)) OR AB ((search strategy or search criteria or systematic search or study selection or data extraction) or (search* N4 literature) or (concept synthesis or conceptual review or critical interpretive synthesis or framework synthesis or integrative review or integrative literature review or literature review or meta-data-analysis or meta-ethnography or (meta N2 narrative) or meta-study or meta-synthesis or mixed method* review or mixed research synthesis or mixed studies review or narrative review or narrative synthesis or realist review or realist synthesis or scoping review or scoping study or qualitative evidence synthesis or qualitative interpretive meta-synthesis or qualitative research synthesis or qualitative systematic review or thematic synthesis or theoretical synthesis))
**PsycINFO**	**(1)** (Cooperation/ or Collaboration/ or Interdisciplinary Treatment Approach/ or Work Teams/ or (collaborati* or cooperati* or co-operati* or crossdisciplinar* or cross-disciplinar* or integrated care or interdisciplin* or inter-disciplin* or interprofession* or inter-profession* or multidisciplin* or multi-disciplin* or multiprofession* or multi-profession* or team* or transdisciplin* or trans-disciplin*).ti,ab,id.)<br>AND<br>**(2)** (Community Health/ or Community Mental Health/ or Community Mental Health Services/ or Home Care/ or Family Medicine/ or General Practitioners/ or Family Physicians/ or Primary Health Care/ or (((community or primary) adj3 (care or health or

	healthcare or practitioner*)) or (community based medicine or (communit$3 adj5 nurse?) or community nursing or community pharmac* or family doctor* or family medicine or family physician* or family medical practice* or family practi* or GP or GPs or general medical practice or general medicine or general physician* or general practi* or health center? or health centre? or medical home* or primary practice)).ti,ab,id.)<br>AND<br>**(3)** (Meta Analysis/ or (meta analy* or metanaly* or metaanaly* or meta regression).ti,ab. or ((systematic* or evidence*) adj3 (review* or overview*)).ti,ab. or (search strategy or search criteria or systematic search or study selection or data extraction).ab. or (search* adj4 literature).ab. or (concept synthesis or conceptual review or critical interpretive synthesis or framework synthesis or integrative review or integrative literature review or literature review or meta-data-analysis or meta-ethnography or (meta adj2 narrative) or meta-study or meta-synthesis or mixed method* review or mixed research synthesis or mixed studies review or narrative review or narrative synthesis or realist review or realist synthesis or scoping review or scoping study or qualitative evidence synthesis or qualitative interpretive meta-synthesis or qualitative research synthesis or qualitative systematic review or thematic synthesis or theoretical synthesis).ti,ab.)
**Cochrane Database of Systematic Reviews + Database of Abstracts of Reviews of Effect (DARE)**	**(1)** ((collaborati* or cooperati* or co-operati* or crossdisciplinar* or cross-disciplinar* or integrated care or interdisciplin* or inter-disciplin* or interprofession* or inter-profession* or multidisciplin* or multi-disciplin* or multiprofession* or multi-profession* or team* or transdisciplin* or trans-disciplin*)<br>AND<br>**(2)** (((community or primary) near/3 (care or health or healthcare or practitioner*)) or (community based medicine or (communit* near/5 nurse?) or community nursing or community pharmac* or family doctor* or family medicine or family physician* or family medical practice* or family practi* or GP or GPs or general medical practice or general medicine or general physician* or general practi* or health center? or health centre? or medical home* or primary practice))):ab,ti
**JBI Database of Systematic Reviews and Implementation Reports**	**(1)** (Cooperative Behavior/ or Interprofessional Relations/ or Physician-Nurse Relations/ or Interdisciplinary Communication/ or Patient Care Team/ or (collaborati* or cooperati* or co-operati* or crossdisciplinar* or cross-disciplinar* or integrated care or interdisciplin* or inter-disciplin* or interprofession* or inter-profession* or multidisciplin* or multi-disciplin* or multiprofession* or multi-profession* or team* or transdisciplin* or trans-disciplin*).ti,hw,sa.)<br>AND<br>**(2)** (Community Health Centers/ or Community Health Services/ or Community Health Nursing/ or Community Mental Health Services/ or Community Pharmacy Services/ or Home Care Services/ or General Practice/ or Family Practice/ or General

	Practitioners/ or Physicians, Family/ or Physicians, Primary Care/ or Primary Health Care/ or Office Visits/ or Primary Care Nursing/ or community health.sa. or (((community or primary) adj3 (care or health or healthcare or practitioner*)) or (community based medicine or (communit$3 adj5 nurse?) or community nursing or community pharmac* or family doctor* or family medicine or family physician* or family medical practice* or family practi* or GP or GPs or general medical practice or general medicine or general physician* or general practi* or health center? or health centre? or medical home* or primary practice)).ti,hw,sa.)<br>AND<br>**(3)** (meta-analysis/ or meta-analysis as topic/ or (meta analy* or metanaly* or metaanaly* or meta regression).ti,tx. or ((systematic* or evidence*) adj3 (review* or overview*)).ti,tx. or (search strategy or search criteria or systematic search or study selection or data extraction).tx. or (search* adj4 literature).tx. or (concept synthesis or conceptual review or critical interpretive synthesis or framework synthesis or integrative review or integrative literature review or literature review or meta-data-analysis or meta-ethnography or (meta adj2 narrative) or meta-study or meta-synthesis or mixed method* review or mixed research synthesis or mixed studies review or narrative review or narrative synthesis or realist review or realist synthesis or scoping review or scoping study or qualitative evidence synthesis or qualitative interpretive meta-synthesis or qualitative research synthesis or qualitative systematic review or thematic synthesis or theoretical synthesis).ti,tx. or ("systematic review protocols" or systematic reviews).pt.)
**PROSPERO**	**(1)** ((collaborati* or cooperati* or co-operati* or crossdisciplinar* or cross-disciplinar* or integrated care or interdisciplin* or inter-disciplin* or interprofession* or inter-profession* or multidisciplin* or multi-disciplin* or multiprofession* or multi-profession* or team* or transdisciplin* or trans-disciplin*)<br>AND<br>**(2)** (community based medicine or community care or community health or community healthcare or community mental health service* or community mental health nursing or community nurse or community nurses or community nursing or community pharmac* or community practitioner* or family doctor* or family medicine or family physician* or family medical practice* or family practi* or GP or GPs or general medical practice or general medicine or general physician* or general practi* or health center or health centers or health centre or health centres or medical home* or primary care or primary health or primary healthcare or primary practice or primary practitioner*)):CM,CS,CT,IV,OP,PA,RQ,SM,TI
**EPISTEMONIKOS**	**(1)** title:(collaborati* or cooperati* or co-operati* or crossdisciplinar* or cross-disciplinar* or integrated care or interdisciplin* or inter-disciplin* or interprofession* or inter-profession* or multidisciplin* or multi-disciplin* or multiprofession* or multi-profession* or team* or transdisciplin* or trans-disciplin*)

	AND<br>**(2)** title:("community based medicine" OR "community care" OR "community health" OR "community healthcare" OR "community mental health service" OR "community mental health services" OR "community mental health nursing" OR "community nurse" OR "community nurses" OR "community nursing" OR "community pharmacy" OR "community pharmacies" OR "community practitioner" OR "community practitioners" OR "family doctor" OR "family doctors" OR "family medicine" OR "family physician" OR "family physicians" OR "family medical practice" OR "family medical practices" OR "family practice" OR "family practitioner" OR "family practitioners" OR GP OR GPs OR "general medical practice" OR "general medicine" OR "general physician" OR "general physicians" OR "general practice" OR "general practitioner" OR "general practitioners" OR "health center" OR "health centers" OR "health centre" OR "health centres" OR "medical home" OR "medical homes" OR "primary care" OR "primary health" OR "primary healthcare" OR "primary practice" OR "primary practitioner" OR "primary practitioners")

Table S2: Data extraction form

**Review details** (indicate page where information was found)	
**Study ID** (First_author Year)	
**Title**	
**Published protocol**	☐ yes *(see attached)* ☐ no
**Review objectives**	
**Type of review**	**A review that seeks to include:**<br>☐ Qualitative studies<br>☐ Quantitative studies<br>☐ Mixed methods studies<br>☐ Conceptual / theoretical studies<br>Other:<br>☐ Not specified
	Type of review, as cited by authors (e.g. "narrative review", "integrative review", "qualitative synthesis"):
**Population of review**	**Restrictions on patients characteristics:**<br>☐ age:<br>☐ condition:<br>☐ gender/ethnicity/other:<br>☐ No restrictions in patient characteristics
**Context / setting of review**	**Definition/description of the primary care setting:**<br><br>**The primary studies of the review includes the following settings:**<br>☐ GP offices / office-based practices (☐ solo, ☐ group)<br>☐ Community health centers<br>☐ Primary health care practices<br>☐ Patient-centered medical home (PCMH)<br>☐ Across settings: interface between primary care and secondary/tertiary/community care/other organizations services<br>☐ "Primary care" (unspecified)<br>☐ "Community care" (unspecified)<br>☐ Other:
	**Geographic boundary of the review:** ☐ No geographic boundary
**Definition of IPC / description of the intervention**	**Definition of IPC** (theoretical and/or operational) **/ of the intervention**:

	**Typology of collaboration**<br>☐ Collaboration within primary care practices/institutions<br>☐ Collaboration between primary care provider(s) and other healthcare professional(s) working outside the primary care sector<br><br>☐ Dyad (e.g. GP-nurse, GP-pharmacist)<br>☐ More than 2 health disciplines
	Notes:
**Outcomes assessed**	☐ Barriers and/or facilitators<br>☐ Effect of IPC on quality of care (process and/or outcome)<br>☐ … at the patient level<br>☐ … at the healthcare professional level<br>☐ … at the organizational level (other than cost)<br>☐ cost-effectiveness<br>☐ Theoretical models, typologies or conceptual frameworks
	More precisely:
**Search strategy and methods**	
**Sources / database searched**	☐ Medline ☐ ProQuest ☐ Lists of references<br>☐ Embase ☐ Web of Science ☐ Grey literature<br>☐ Cinahl ☐ Scopus ☐ Other:<br>☐ PsycINFO ☐ Pascal ☐ Other:<br>☐ CENTRAL ☐ Cochrane library ☐ Other:<br>☐ HealthSTAR ☐ JBI EBP database ☐ Other:
**Search restrictions** (language, years, region, etc.)	
**Other eligibility criteria**	
**Instrument / tool used for quality appraisal of studies**	
***Characteristics of included studies* table**	☐ Yes: Table n°:<br>Additional file n°:<br>☐ No
**Method of analysis / synthesis of results**	☐ Narrative synthesis:<br>☐ Thematic analysis:<br>☐ Taxonomic analysis:<br><br>Presentation of results supported by:<br>☐ Tabulation<br>☐ Framework/model

	☐ Other qual. analysis:<br>☐ Meta-analysis:<br>☐ Other quant. analysis:	☐ Forest plot<br>☐ Other:
**Details of primary studies**		
**Study selection**	Number of records screened:<br>Number of records excluded with reasons:<br>Number of studies included (qualitative analysis):<br>Number of studies included (quantitative analysis):<br>Flow diagram: ☐ Yes, Figure n°:<br>☐ No	
**Range of included studies** (years)		
**Study design of included studies**	☐ Qualitative studies ☐ Mixed methods studies<br>☐ Quantitative studies ☐ Conceptual / theoretical studies<br><br>☐ RCT ☐ ITS ☐ QUAL: Focus groups<br>☐ Q-RCT / NRCT ☐ XS ☐ QUAL: Interviews<br>☐ CBA / BA ☐ CC ☐ QUAL: Observations<br>☐ QUANT/QUAL: Survey<br>☐ Other:	
**Health professionals included in IPC** (from primary studies description)	**Primary care providers**<br>☐ primary care physicians (PCP):<br>☐ family/general practitioners<br>☐ internists<br>☐ pediatricians<br>☐ geriatricians<br>☐ physician assistants<br>☐ other(s):<br>☐ primary care nurses:<br>☐ nurse practitioners<br>☐ practice nurses<br>☐ other(s):<br><br>**Other healthcare professionals**<br>☐ specialized physicians<br>☐ pharmacists<br>☐ physiotherapists<br>☐ occupational therapists<br>☐ dieticians<br>☐ midwives<br>☐ public health practitioners<br>☐ public health nurses<br>☐ community nurses<br>☐ home care nurses<br>☐ social workers<br>☐ psychologist<br>☐ mental health workers<br>☐ community psychiatric nurses<br>☐ receptionists<br>☐ administrative staff<br>☐ practice managers<br>☐ case or clinical managers<br>☐ health navigators<br>☐ health visitors<br>☐ other(s):	

**Comparison groups**	☐ Usual care<br>☐ Other intervention:	☐ Not applicable
**Location of included studies** (e.g. city or region, country)		
**Overall quality appraisal of included studies**		
**Main results**		
**Effectiveness of IPC:**<br>***patient level***		
**Effectiveness of IPC:**<br>***healthcare professional level***		
**Effectiveness of IPC:**<br>***organisation level (other than COST)***		
**COST-effectiveness**		
**Factors (e.g. intervention characteristics) associated with effectiveness**		
**Barriers**		
**Facilitators**		
**Description of models of collaboration**		
**Theoretical model, typology or conceptual framework**		
**Other(s) outcomes/results**		
**Limitations**		

**Other**	
Quality appraisal (ROBIS)	☐Low ☐ High ☐ Unclear
Comments	

Table S3: Characteristics of included reviews on effectiveness of IPC on patient outcomes (n=34)

Author, year	Type of review; Synthesis method	Primary studies details: Number of included studies; Year range; Countries	Intervention	Restric-tion(s) on population or setting	Type of results					Quality assessment (ROBIS)				
					Patient	HCP	Organiza-tional	Cost	Factors	1.	2.	3.	4.	Risk of bias in the review
**Interprofessional collaboration in primary care (large scope)**														
Barrett, 2007	Mixed methods review<br>Narrative synthesis	n=206<br>NA<br>NA	Interprofessional collaboration (two or more different professions)	-	✓	✓	✓	✓		Low	High	High	High	High
DeLoach, 2018	Mixed methods review<br>Narrative synthesis	n=5<br>2012-2018<br>NA	Interprofessional collaboration for the management of type 2 diabetes care in primary care settings	Patients with type 2 diabetes	✓				✓	Unclear	High	Unclear	High	High
Gougeon, 2017	Quantitative review<br>Narrative synthesis	n=6<br>2006-2014<br>Canada (Ontario, Quebec and British Columbia)	Community-based interprofessional teams (at least two different disciplines)	Patients ≥65 living in the community	✓			✓	✓	Low	High	Low	Low	High

Author, year	Type of review; Synthesis method	Primary studies details: Number of included studies; Year range; Countries	Intervention	Restric-tion(s) on population or setting	Type of results					Quality assessment (ROBIS)				
					Patient	HCP	Organiza-tional	Cost	Factors	1.	2.	3.	4.	Risk of bias in the review
Martin, 2010	Quantitative review Narrative synthesis	n=14 1999-2008 USA, England, Germany, Netherlands, Australia, Canada	Care management interventions with interprofessional collaboration (two or more different professions, including doctors and nurses)	-	✓					High	High	High	High	High
Proia, 2014	Quantitative review Narrative synthesis with descriptive statistics	n=52 2003-2011 US, Europe, Canada, Japan	Team-based care involving a primary care provider and other professionals	Patients with hypertensi on	✓				✓	Low	High	Low	Low	High

Author, year	Type of review; Synthesis method	Primary studies details: Number of included studies; Year range; Countries	Intervention	Restric-tion(s) on population or setting	Type of results					Quality assessment (ROBIS)				
					Patient	HCP	Organiza-tional	Cost	Factors	1.	2.	3.	4.	Risk of bias in the review
Schepman, 2015	Quantitative review<br>Coding and pooling of outcomes based on the Donabedian model	n=51<br>1993-2014<br>USA, Canada, UK, Netherlands, Sweden, Belgium, New Zealand, France, Spain, Israel, Australia, Puerto Rico	Multidisciplinary collaboration in primary care and between primary care and other sectors	-	✓				✓	Low	Low	Unclear	High	High
Trivedi, 2013	Quantitative review<br>Narrative synthesis	n=37<br>1990-2008<br>USA, Canada, Switzerland, Norway, Finland, Netherlands, Sweden, Germany, Italy, UK, Hong Kong, Australia	Inter-professional working models (e.g. case management, integrated team)	Communit y-dwelling patients ≥ 65 with long-term conditions	✓			✓		Low	High	High	Low	High

Author, year	Type of review; Synthesis method	Primary studies details: Number of included studies; Year range; Countries	Intervention	Restric-tion(s) on population or setting	Type of results					Quality assessment (ROBIS)				
					Patient	HCP	Organiza-tional	Cost	Factors	1.	2.	3.	4.	Risk of bias in the review
Wranik, 2019	Mixed methods review Narrative synthesis based on an analytical framework	n=77 2003-2017 Canada, Australia, UK, New Zealand	Interprofessional primary care teams	-	✓	✓			✓	Low	Low	Low	Low	Low
**Primary care physician – nurse practitioner collaboration**														
Norful, 2019	Quantitative review Narrative synthesis	n=6 2003-2014 USA	Nurse practitioners-physician teams (co-management)	-	✓					Low	Low	Unclear	Low	Low
**Primary care provider – specialty care provider collaboration**														
Carmont, 2017	Mixed methods review Narrative synthesis	n=17 2001-2014 Australia, UK, Canada, Denmark, Netherlands, unspecified	Integration/ shared care between GP[a] and specialist secondary clinician/ service in palliative care	Palliative care	✓	✓				Low	High	Low	Low	Low

Author, year	Type of review; Synthesis method	Primary studies details: Number of included studies; Year range; Countries	Intervention	Restric-tion(s) on population or setting	Type of results					Quality assessment (ROBIS)				
					Patient	HCP	Organiza-tional	Cost	Factors	1.	2.	3.	4.	Risk of bias in the review
Foy, 2010	Quantitative review Meta-analysis	n=23 1992-2008 USA, Netherlands, Canada, UK, Australia, France, Austria, Israel	Interactive communication (two-way) between primary care physicians and specialists	Psychiatry and endocrino-logy care	✓				✓	Low	Low	Low	Low	Low
Mitchell, 2002	Quantitative review Narrative synthesis	n=7 1994-1999 NA	Interventions involving organized cooperation between GP[a] and specialists	-	✓	✓		✓		Low	High	High	High	High
Mitchell, 2015	Quantitative review Narrative synthesis	n=10 2002-2010 New Zealand, Australia, UK, US, Belgium, Ireland	Integrated models delivered at the primary-secondary interface (with direct interaction between primary and secondary care providers)	Adults patients with chronic conditions	✓	✓		✓		High	Low	High	High	High

Author, year	Type of review; Synthesis method	Primary studies details: Number of included studies; Year range; Countries	Intervention	Restric-tion(s) on population or setting	Type of results					Quality assessment (ROBIS)				
					Patient	HCP	Organiza-tional	Cost	Factors	1.	2.	3.	4.	Risk of bias in the review
Smith, 2017	Quantitative review Meta-analysis	n=42/22[2] 1992-2015 UK, USA, Australia, New Zealand, Netherlands, Denmark, Belgium, Ireland, Sweden, Spain, Italy and Puerto Rico	Shared care/ integrated care models between primary care and specialty care physicians	Patients with chronic conditions	✓			✓		Low	Low	Low	Low	Low
**Primary care provider – pharmacist collaboration**														

**Author, year**	**Type of review; Synthesis method**	**Primary studies details: Number of included studies; Year range; Countries**	**Intervention**	**Restric-tion(s) on population or setting**	**Type of results**					**Quality assessment (ROBIS)**				
					**Patient**	**HCP**	**Organiza-tional**	**Cost**	**Factors**	**1.**	**2.**	**3.**	**4.**	**Risk of bias in the review**
Hazen, 2017	Quantitative review<br>Narrative synthesis with descriptive statistics	n=60<br>1996-2015<br>USA, UK, Canada, Brazil, Jordan, Australia, Sweden, Hong Kong	Clinical pharmacy services aimed at improving medication use conducted by a non-dispensing clinical pharmacist (partly) co-located in a primary care team	-	✓				✓	Low	Low	Low	Unclear	Unclear
Kwint, 2013	Quantitative review<br>Meta-regression analysis	n=12<br>2001-2011<br>US, UK, Netherlands, New Zealand, Canada, Australia	Medication review interventions involving pharmacists and GP[a]	Home-dwellling patients ≥70	✓				✓	High	High	Low	Low	High

Author, year	Type of review; Synthesis method	Primary studies details: Number of included studies; Year range; Countries	Intervention	Restric-tion(s) on population or setting	Type of results					Quality assessment (ROBIS)				
					Patient	HCP	Organiza-tional	Cost	Factors	1.	2.	3.	4.	Risk of bias in the review
Health Quality Ontario, 2009	Quantitative review Meta-analysis	n=8[b] 2005-2008 USA, Canada	Multidisciplinary community care intervention for the management of type 2 diabetes involving at least a pharmacist and a primary care physician	Adult patients with diabetes	✓					Unclear	High	Unclear	Unclear	Unclear
**Primary care provider-mental healthcare provider collaboration ("Collaborative care")**														
Archer, 2012	Quantitative review Meta-analysis	n=79 1993-2011 US, UK, Germany, Netherlands, Canada, Chile, India, Puerto Rico.	"Collaborative care" for depression and anxiety	Patients with depression or anxiety	✓					Low	Low	Low	Low	Low

**Author, year**	**Type of review; Synthesis method**	**Primary studies details: Number of included studies; Year range; Countries**	**Intervention**	**Restric-tion(s) on population or setting**	**Type of results**					**Quality assessment (ROBIS)**				
					**Patient**	**HCP**	**Organiza-tional**	**Cost**	**Factors**	**1.**	**2.**	**3.**	**4.**	**Risk of bias in the review**
Bower, 2006	Quantitative review<br>Meta-analysis	n=34<br>1993-2004<br>USA, UK, Sweden, Chile, The Netherlands	"Collaborative care" for depression	Patients with depression	✓				✓	Low	Unclear	Unclear	Unclear	Unclear
Butler, 2008	Quantitative review<br>Narrative synthesis	n=33<br>1992-2007<br>US	Integration of mental health services into primary care settings	Patients with mental health disorder or alcohol related substance abuse	✓			✓	✓	Low	Low	Low	High	Low
Coventry, 2014	Quantitative review<br>Meta-analysis	n=74<br>1993-2013<br>US, UK, Canada, Chile, Germany, India, Netherlands, Puerto Rico	"Collaborative care" for depression	Patients with depression	✓				✓	Low	High	High	High	High

Author, year	Type of review; Synthesis method	Primary studies details: Number of included studies; Year range; Countries	Intervention	Restric-tion(s) on population or setting	Type of results					Quality assessment (ROBIS)				
					Patient	HCP	Organiza-tional	Cost	Factors	1.	2.	3.	4.	Risk of bias in the review
Craven, 2006	Quantitative review Narrative synthesis	n=38 1992-2005 US, UK, Australia, unspecified	Collaboration between primary care providers and mental health providers	-	✓				✓	Low	High	High	High	High
Dham, 2017	Mixed methods review Narrative synthesis	n=29 1994-2016 USA, UK, Australia, Canada	"Collaborative care" for psychiatric disorder(s)	Older adults with psychiatric disorder(s)	✓			✓		Low	High	High	High	High
Fuller, 2011 a	Mixed methods review Narrative synthesis	n=42 1998-2009 UK, USA, Australia, Canada, Anguilla, EEC, New-Zealand	Primary mental health care linkage (connection between two or more professionals, including a primary care provider)	Primary mental health care	✓			✓	✓	Low	Low	High	High	High

Author, year	Type of review; Synthesis method	Primary studies details: Number of included studies; Year range; Countries	Intervention	Restric-tion(s) on population or setting	Type of results					Quality assessment (ROBIS)				
					Patient	HCP	Organiza-tional	Cost	Factors	1.	2.	3.	4.	Risk of bias in the review
Gilbody, 2006	Quantitative review Meta-analysis	n=37 1993-2006 USA, UK, Sweden, Chile, The Netherlands	"Collaborative care" for depression	Patients with depression	✓				✓	Low	Unclear	High	Low	Unclear
Gunn, 2006	Quantitative review Narrative synthesis	n=11 1996-2004 USA, UK	Multi-professional complex interventions for depression in primary care	Adult patients with depression	✓					Low	High	Low	Low	Low
Huang, 2013	Quantitative review Meta-analysis	n=10/8[c] 2004-2012 US	"Collaborative care" for both depression and diabetes	Patients with both depression and diabetes	✓					Low	Low	Low	High	Low
Muntingh, 2016	Quantitative review Meta-analysis	n=7 2001-2014 US, Netherlands, Germany	"Collaborative care" for anxiety	Adult patients with anxiety	✓				✓	Low	Low	Low	Low	Low

Author, year	Type of review; Synthesis method	Primary studies details: Number of included studies; Year range; Countries	Intervention	Restric-tion(s) on population or setting	Type of results					Quality assessment (ROBIS)				
					Patient	HCP	Organiza-tional	Cost	Factors	1.	2.	3.	4.	Risk of bias in the review
Panagioti, 2016	Quantitative review Meta-analysis	n=31 1995-2015 US, Europe, Canada, India	"Collaborative care" for depression	Adult patients with depression	✓				✓	Low	Low	Unclear	Low	Low
Sighinolfi, 2014	Quantitative review Meta-analysis	n=17 1995-2013 UK, The Netherlands, Italy Germany, Spain	"Collaborative care" for depression	Adult patients with depression	✓				✓	Low	Low	Low	Low	Low
Thota, 2012	Quantitative review Meta-analysis	n=32 2004-2009 US, England, Canada, Germany, Australia, The Netherlands, Switzerland, Scotland	"Collaborative care" for depression	Patients with depression	✓				✓	Low	Unclear	Low	Low	Low

Author, year	Type of review; Synthesis method	Primary studies details: Number of included studies; Year range; Countries	Intervention	Restric-tion(s) on population or setting	Type of results					Quality assessment (ROBIS)				
					Patient	HCP	Organiza-tional	Cost	Factors	1.	2.	3.	4.	Risk of bias in the review
van Steenbergen-Weijenburg, 2010	Quantitative review Narrative synthesis	n=8 1998-2007 US, Chile	"Collaborative care" for depression	Patients with depression	✓			✓		Low	High	Unclear	High	High
**Intersectoral collaboration (nursing home, public health)**														
Davies, 2011	Mixed methods review Narrative synthesis, framework analysis	n=17 1998-2008 UK, Australia, USA, Sweden	Integration between health care professionals and care home staff	Nursing homes	✓					Low	Low	Low	Low	Low
Martin-Misener, 2012	Mixed methods review Thematic analysis	n=114 1989-2008 UK, USA, Canada, Australia, New Zealand, Finland and international papers	Collaboration between primary care and public health (interorganizational collaboration)	-	✓	✓	✓	✓		Low	Low	High	High	High

Author, year	Type of review; Synthesis method	Primary studies details: Number of included studies; Year range; Countries	Intervention	Restric-tion(s) on population or setting	Type of results					Quality assessment (ROBIS)				
					Patient	HCP	Organiza-tional	Cost	Factors	1.	2.	3.	4.	Risk of bias in the review

[a]GP: General practitioners
[b] Extraction of the results of 3 out of 8 studies (related only to research question n°2)
[c] Qualitative synthesis/meta-analysis

Table S4: Detailed results of included reviews for patient outcomes[a] (n=34)

Author, year	Effectiveness for patient outcomes and associated factors
**Interprofessional collaboration in primary care (large scope)**	
Barrett, 2007	**IPC approach (versus uniprofessional model):**<br>- More satisfaction, positive experience and perception of care<br>- Developing enhanced self-care and health condition knowledge and skills<br>- Different health practices (for example, improved self-care, lifestyle and preventive service access)<br>- better health outcomes (for example, blood pressure control, diabetes control, health status, quality of life)
DeLoach, 2018	- Pharmacists' interventions that impacted the cardiovascular disease risk factor of BMI included medication management, patient education, and feedback to physicians: significant reduction of BMI (weighted mean difference -0.9kg/m2 [-1.7 to-0.1], p = 0.026) (one study)<br>- Integration of pharmacists in IPC teams: statistically and clinically significant reduction in HA1c of an average of 1.2% (p < .001), unchanged BMI (one study)<br>- Health care processes including equal and comprehensive involvement with physicians, nurses, and dieticians: fewer patients with higher HA1c percentages; when there was no designated leader: lower percentage of patients who showed no improvement or maintained a HA1c measurement of over 9% when compared to the total population (3% vs. 16%, p = 0.03)<br>- The development of a facilitator-led care coordination project including cross-discipline discussions and the development of shared action plans designed to contact and assist patients who did not show an improvement in their health outcomes: decrease of average HA1c from 10.6% to 8.8%<br>- Use of information systems (such as EHR) as a tool for patient identification, registry and monitoring and/or to enhance communication
Gougeon, 2017	- IP teams have greater positive effects on patient-reported measures of health (distal measures, e.g., increased satisfaction with care and quality of life) than direct measures of health (e.g., decreased visits to the emergency and hospitalization)<br>- Effect size: larger and longer interventions reported more desirable outcomes<br>- Fully collaborative IP models tended to report greater number of favorable outcomes with greater effect sizes compared to partially collaborative models (limited number of studies)
Martin, 2010	Intervention versus usual care/ control group<br>- No difference in mortality (5 studies)<br>- 49% reduction in all-cause mortality during the second year (odds ratio: 0.51, 95% CI:0.29–0.91, p = 0.02) (1 study)

	- Median survival in patients with chronic heart failure almost two times higher (40 versus 22 months respectively, p <0.001) and fewer deaths overall (adjusted relative risk: 0.74, 95% CI: 0.53–0.80, p <0.001) after up to ten years' follow-up (1 study)<br>- Improvement in physical, emotional or social functioning (5 studies), others showed no differences (4 studies)<br>- Significant reduction in medical service use (5 studies), others showed mixed results (2 studies)<br>- Significant reduction in emergency department visits without hospitalization in low-income elderly population, but no differences for hospital admission rates (1 study)<br>- Trend towards lower hospitalization rates for any reason among psychiatric patients during 2nd year of study, significant reduction in 3rd year (34 % versus 48%, respectively, p = 0.02) (1 study)<br>- No differences regarding hospitalization rates or length of hospital stay (2 studies)<br>- Geriatric intervention group: significantly higher score of self-perceived health and life satisfaction (p = 0.04) (1 study)<br>- Chronically ill seniors: increase in social activities compared with decrease in the control group (p = 0.04) (1 study)<br>- No differences in activities of daily living (2 studies), whereas one study showed significant improvement in functional abilities during the first 6 months; after, effects were no longer significant<br>- More patient satisfaction (4 studies)
Proia, 2014	-Median effect estimate of 12 pct pts for proportion of patients with controlled BP; most individual effect estimates in the favorable direction were significant (p<0.05) (33 studies)<br>- Median reduction in SBP of 5.4 mmHg; most individual effect estimates were significant (p<0.05) (44 studies)<br>- Overall median reduction in DBP of 1.8 mmHg (38 studies)<br>- Three studies not included in main analyses: groups receiving team- based care had poorer BP outcomes than those receiving usual care (p>0.05) (1 study), high- income patients were significantly more likely to have BP control with team-based care (p<0.05) (1 study), slight improvements in BP control (p=0.23) and SBP (p=0.12) and no change in DBP (p=0.37) for patients receiving home health visits (1 study).<br>- Significant decreases in both myocardial infarctions and any CVD-related event were reported (OR=0.24 and 0.47, respectively, p < 0.05); team-based care group had 25 deaths compared to 36 deaths in the control group (OR=0.55, p < 0.05) after 12 months (1 study)<br>- Proportion of patients receiving team-based care with "high" medication adherence increased by median of 16.3 pct pts<br>- Satisfaction with care at 12 months: high patient satisfaction scores for hyper-tension care in both the team-based and usual care groups (p = 0.75) with no significant association between satisfaction and BP goal achievement (p=0.40) (1 study); improvement of 14.0 pct pts in proportion of patients reporting satisfaction with team-based care approach (p<0.001) (1 study)<br>- Team-based care: improvements for most lipid- and diabetes-related outcomes, suggesting potential benefits for comprehensive CVD risk reduction (22 studies); reduction in depressive symptoms (2 studies)<br>- Improvement in the proportion of patients with controlled BP was similar for studies from both healthcare and community settings.

	- Median effect estimate from studies within the Veterans Affairs (VA) system was 5.9 pct pts<br>- Pharmacists: median improvement in proportion of patients with controlled BP was considerably higher than overall estimate, but median reductions in SBP and DBP were similar to overall estimates<br>- Nurses alone or both nurses and pharmacists: median estimates for all BP outcomes comparable to overall estimates<br>- Other team members (such as community health workers, integrated care managers, etc.): smaller effect estimates than overall<br>- Team member involvement in medication management – 3 levels: (1) make changes to medications independent of the primary care provider (16 studies); (2) provide medication recommendations and make changes with the primary care provider's approval (15 studies); or (3) only provided adherence support and information on medication and hypertension (22 studies); first 2 levels of medication management led to larger improvements in BP outcomes than overall estimates (1 study)<br>- Adding two or more members: larger improvements in proportion of patients with controlled BP and reduction in DBP compared to adding only one; median reductions in SBP similar regardless of team size<br>- Larger improvements for patients when < 50% of these had controlled BP at baseline (14 studies)<br>- Patients with average SBP > 140 mmHg at baseline had greater reduction in SBP (26 studies); similar trend for patients with an average DBP reading > 90 mmHg at baseline (6 studies)
Schepman, 2015	- Proportion of significant outcomes is lower than 50% (51 studies); 16 studies showed no significant outcomes<br>- Of the 139 outcomes (all studies): aprox. half were non-significant; 38% of patient-reported outcomes showed significant positive effects; 54% of clinical outcomes significant, 46% of use of health services outcomes significant; 47% of professional-reported outcomes significant<br>- Clinical outcomes: most frequently positive and significant (54%)<br>- No significant difference between interventions with or without physician involved in the collaboration<br>- Medium and large interventions (number of disciplines involved): no relation with positive or statistically significant effects for each type of outcome<br>- Smaller collaborations: proportion of clinical outcomes most likely to be significant (not tested)<br>- Non-specific age groups more often show positive significant outcome (p < .05); only 'use of health services' reported higher proportion of positive and significant outcomes for interventions targeted at older people<br>- Collaborations directed towards older patients: less positive significant clinical and patient-related outcomes<br>- Interventions for patients with physical or multiple physical chronic conditions: more significant clinical outcomes (not tested)<br>- Care coordination interventions: highest proportion of significant effects on outcomes<br>- Individual care plans: most often report positive clinical outcomes<br>- Collaborations between primary care and providers from other sectors: no significant difference from other collaborations
Trivedi, 2013	- Properly integrated and shared care models improved processes of care with a potential to diminish care use

	- Case Management (7 studies): 4 studies described chronic care, 1 palliative care and 2 preventive home care with mixed evidence of effect; 4 showed some improvement in health outcomes, most improved patient/ user satisfaction<br>- Collaboration (11 studies): 3 focused on acute care, 4 described chronic care, 3 preventive home-based care and 1 outpatient care. Around half reported improved health/ functional outcomes; most detecting improved process measures and patient/ user satisfaction.<br>- Integrated Team (19 studies): many showed improved health/ functional ability, reduced caregiver burden, user satisfaction and process measures, including quality of care
Wranik, 2019	- Logic model: Team Characteristics > Care and Team Processes > Health Care Outputs > Health Outcomes<br>- Addition of specific professions to teams: moderate evidence of improved care processes (expansion of nursing role improved care coordination, satisfaction of providers and patients; addition of pharmacists more challenging)<br>- Addition of new providers and new models of care: strong quantitative evidence of associated with increases in provision of recommended tests and preventive services, and decrease in use of hospitals<br>- Inclusion of new types of providers (e.g. pharmacist, a nurse practitioner or a nurse): mixed quantitative evidence of association with improved health markers<br>- Addition of pharmacists: moderate, conflicting evidence, of effect on appropriateness of medications used by patients<br>- Addition of practice nurses: strong qualitative and quantitative evidence of positive impact on provision of recommended tests, screens, and preventive services<br>- Addition of new types of health care providers: strong qualitative evidence of expansion of range of services
**Primary care physician – nurse practitioner collaboration**	
Norful, 2019	-Significantly more recommended care guidelines completed (e.g. exams and screening performed, test offered, treatment offered, behavioral interventions instituted)<br>-Mixed results for clinical outcomes and quality of life
**Primary care provider – specialty care provider collaboration**	
Carmont, 2017	- GP engagement in palliative care with relevant specialized secondary services: effective and positive outcomes for hospital (reduced readmissions, p=0.0069; p=0.001; shortened length of stay)<br>- GP engagement with secondary services in integrated care at the end of life, case conferences, home conferences and good practice framework: improvements in communication, relationships between services, professional development and patient-perceived benefits (pain management, symptom control, security)
Foy, 2010	- Mental health RCTs: improvement of 4.6 points on CES-D depression scale (SMD -0.41, 95% CI,-0.73 to -0.10) (n=11)<br>- Mental health non-RCTs: improvement of 5.3 points on CES-D depression scale (SMD -0.47, 95% CI,-0.84 to -0.09) (n=7)<br>- Diabetes non-RCTs: improvement of 1.4% in HbA1c (SMD -0.64, 95% CI,-0.93 to -0.34) (n=5)

	- Meta-regression: studies with interventions to improve quality of information exchange (such as structured forms, pathways, or reports) had statistically and clinically significant benefits in outcomes
Mitchell, 2002	- Process of care outcomes: improvements in retention in programs involving GP for patient with hypertension, diabetes and chronic schizophrenia (3 studies)<br>- Patient satisfaction: GP involvement led to improvements for patients with diabetes, hypertension, chronic schizophrenia and geriatric problems (4 studies)<br>- Studies addressed very different illness groups
Mitchell, 2015	- Many clinical outcomes: no difference<br>- Service utilization outcomes: mixed results (improvements in some studies and deterioration in others)
Smith, 2017	- No consistent improvement in any type of patient outcome could be observed<br>- Medication related outcomes: modest effect; for depression, medication appropriateness and adherence in favor of shared care; lacking evidence for other chronic conditions
**Primary care provider – pharmacist collaboration**	
Hazen, 2017	- No association between degree of integration and improvement of health outcomes<br>- Association between degree of integration and improvement of health outcomes: negative association (high integration = worse health outcomes) for disease-specific pharmacy services (e.g. diabetes, COPD); positive association (high integration = better health outcomes) for patient-centered pharmacy services (e.g. polypharmacy)
Kwint, 2013	- Number of key elements present in the intervention (more intensive GP-pharmacist collaboration in medication review, e.g. follow-up by care provider, pharmacist with clinical experience, access to medical records): significant positive association with implementation rate of recommendations (defined as percentage of recommendations arising from medication review, e.g. decrease in the number of potentially inappropriate prescriptions, PIPs, fully or partly implemented and/or the percentage of DRPs resolved), $\beta = 0.085$ (95 % CI 0.052–0.128; $p < 0.0001$); no association with QoL, functioning, other PROMs or service utilization outcomes.<br>- Process of care outcomes (e.g. number of drugs prescribed): mixed results<br>-Pharmacists working at a GP practice: high rates of acceptance of recommendations
Health Quality Ontario, 2009	- Statistically and clinically significant reduction in HbA1c and in SBP (meta-analysis on 2 RCTs)
**Primary care provider – mental health care provider collaboration ("Collaborative care")**	
Archer, 2012	- CC for patients (adults) on clinical outcomes: more effective than usual care for depression at 6 (SMD -0.34, 95% CI -0.41 to -0.27; RR 1.32, 95% CI 1.22 to 1.43), 12 (SMD -0.28, 95% CI -0.41 to -0.15; RR 1.31, 95% CI 1.17 to 1.48) and 24 months (SMD -

	0.35, 95% CI -0.46 to - 0.24; RR 1.29, 95% CI 1.18 to 1.41); not significant after (RR 1.12, 95% CI 0.98 to 1.27); more effective than usual care for anxiety at 6, 12, and 24 months.<br>- CC on medication use: antidepressant use increased at 6, 12, and 24 months but not significant after; increase at 12 months for anxiety<br>- CC on QoL, functioning & PROMs & patient satisfaction: more effective than usual care in terms of mental health QoL at 6 (SMD -0.30, 95% CI -0.44 to -0.17; RR 1.50, 95% CI 1.21 to 1.87), 12 (SMD -0.33, 95% CI -0.47 to -0.19; RR 1.41, 95% CI 1.18 to 1.69) and 24 months (SMD -0.20, 95% CI -0.34 to -0.06; RR 1.26, 95% CI 1.11 to 1.42), for physical health quality of life at 24 months, and for patient satisfaction post-intervention
Bower, 2006	- CC: positive effect on antidepressant use (odds ratio 1.92, 95% CI 1.54–2.39) and depressive outcomes (standardised mean difference 0.24, 95% CI 0.17–0.32)<br>- Non-US studies (P=0.038), recruiting through systematic identification of patients (P=0.081), using case managers having a specific mental health background (P=0.027) who received regular supervision (P=0.055) were more effective
Butler, 2008	- The studies reviewed tended to show positive results for symptom severity, treatment response, and remission<br>- Wide variation in the levels of provider integration and integrated processes of care<br>- No clear patterns in Forest plots to suggest that outcomes improve as the levels of either provider integration or integrated process of care increase - Significant improvements in symptom severity, treatment response, and remission consistent across integration levels
Coventry, 2014	- Meta-regression, multivariate analysis (multivariable meta-regression model): CC including psychological interventions predicted improvement in depression (β coefficient -0.11, 95% CI -0.20 to -0.01, p = 0.03).<br>- Systematic identification of patients with depression (relative risk 1.43, 95% CI 1.12 to 1.81, p = 0.004) and presence of a chronic physical condition (relative risk 1.32, 95% CI 1.05 to 1.65, p = 0.02) predicted use of anti-depressant medication<br>- CC including psychological interventions (alone or with medication management) as part of CC were associated with greater improvements in depressive symptoms compared with studies that only included medication management alone<br>- Use of antidepressants was increased in studies that included participants with a chronic physical health condition and in studies that recruited participants through a process of systematic identification
Craven, 2006	- Mixed results for all patient outcomes. Heterogeneity of included studies (condition, context, etc.)<br>- No trend identified between the degree of collaboration and clinical outcomes<br>- Co-location of mental health and primary care providers produce greater engagement of patients in their mental health care<br>- Pairing of collaboration & treatment guidelines benefits patient outcomes with depressive disorders, and the more severe the disorder, the higher the benefit<br>- Systematic follow-up consistent with treatment guidelines and including mechanism to alter treatment if patients weren't responding well was the most powerful predictor for positive clinical outcomes

	- Enhanced patient education on mental health disorders and their treatment (by health professional other that PCP) benefitted patient outcomes<br>- Patient choice in treatment modality enhances engagement in collaborative care
Dham, 2017	- Variability depending on condition and setting (leading to variability in implementation of IPC)<br>- Mixed results for clinical outcomes, QoL, functioning and PROMs, and patient satisfaction<br>- Improvement for medication outcomes (treatment initiation and adherence for depression), for process of care outcomes (client engagement with number of visits, greater adherence to recommended care guidelines for dementia)
Fuller, 2011a	- 23 statistically significant positive clinical outcomes out of 37<br>- Depression trials provided the major evidence of clinical effectiveness (12 trials) (with/without dysthymia and associated alcohol risk): symptom reduction, reduced severity, better treatment response, and improvements in physical and social functioning<br>- 15 statistically significant positive delivery outcomes out of 25<br>- Depression trials showed significant positive service delivery effect (10 trials of depression or dysthymia and one with associated alcohol risk).<br>- Depression trials included improvements in service delivery (targeted referrals, reduced rates of hospitalization and patient engagement with treatment, such as increased use of and self-efficacy with appropriate medication and adherence to other treatments)<br><br>**Sub-group analysis:**<br>- Patients with chronic and complex psychotic illnesses: some clinical benefit reported (such as improved mental and physical function with the use of a case manager, improved physical function with an integrated clinic and reduced relapse with a quality program to improve team communication<br>- Some improved communication between co-located services and increased referral to mental health services, other study (cohort) showed no improvement in clinical outcomes or service use<br>- Successful collaborative clinical programs in primary mental health care use multiple linkages that impact on the direct work of clinicians, more so than on management level agreement across services<br>- A lower proportion of studies that used linkages from a single broad category showed positive outcomes, compared to those studies that used linkages from multiple broad categories<br>- Most common linkages in studies with a positive effect were care management, enhanced communication, consultation liaison and local protocols<br>- Linkages mostly involved the following components: the primary care physician received screening or diagnosis results; patients were notified of screening results; guideline specific treatment was promoted including annual screening with or without a treatment plan; other clinical information was provided; or patients could self-refer to the mental health services

Gilbody, 2006	- CC had a positive effect on standardized depression outcomes at 6 months compared with standard care (SMD, 0.25; 95% CI, 0.18-0.32); moderate level of heterogeneity between studies (I^2=52.8%)<br>- Clinical improvement to be maintained at 12 (SMD, 0.31; 95% CI, 0.01 to 0.53), 18 (SMD, 0.25; 95% CI, 0.03 to 0.46), 24 months (SMD, 0.15; 95% CI, −0.03 to 0.34), and 5 years (SMD, 0.15; 95% CI, 0.001 to 0.30), not statistically significant at 24 months (11 studies); substantial between-study heterogeneity (I^2=84% at 24 months)<br>- Compliance with medication predicted depression outcomes with credible certainty (slope coefficient, 0.19; 95% credible interval, 0.08-0.30).<br>- Studies from the US showed a strongly positive and statistically significant effect for CC in improving depression outcomes at 6 months ($SMD_{US\ studies}$, 0.27; 95% CI, 0.22-0.33); minimal between-study heterogeneity (I^2US studies, 5.4%).<br>- Use of regular and planned supervision of the case manager, usually by a psychiatrist, was related to a more positive clinical outcome ($SMD_{usual\ supervision}$, 0.29; $SMD_{unplanned\ and\ ad\ hoc\ supervision}$, 0.14; meta-regression β, 0.15; 95% CI, −0.02 to 0.31; P=.07; I^2= 49.3%).<br>- Case managers with a specific mental health background also achieved better outcomes ($SMD_{CMmental\ health\ background}$, 0.34; $SMD_{CMnon–mental\ health\ background}$, 0.164; meta-regression, 0.18; 95% CI, 0.04-0.32; P=.02; I^2=42.4%).
Gunn, 2006	- Interventions implemented in US for patients willing to take antidepressant medication show a modest increase in recovery from depression<br>- 8 trials showed an increase in recovery for patients in intervention group (range from 10% to 33%); (attrition rates from 5 to 50%)<br>- Trials reported outcomes at varying time-points from 3, 4, 6, 12, 24 to 57 months<br>- Four trials reported recovery outcomes at or beyond one year of follow-up, with three of these reporting findings in favor of the intervention
Huang, 2013	- CC showed a significant improvement in depression treatment response (RR = 1.33, 95% CI = 1.05-1.68), depression remission (adjusted RR = 1.53, 95% CI =1.11-2.12), higher rates of adherence to antidepressant medication (RR = 1.79, 95% CI = 1.19-2.69) and oral hypoglycemic agent (RR = 2.18, 95% CI = 1.61-2.96), but a non-significant reduction in HbA1c values (MD = -0.13, 95% CI = -0.46-0.19)
Muntingh, 2016	- CC was superior to care as usual, with a small effect size (SMD = 0.35 95 % CI 0.14–0.56) for all anxiety disorders combined and a moderate effect size (SMD = 0.59, 95 % CI 0.41–0.78) in a subgroup analysis (five studies) on patients with panic disorder.<br>- Studies performed in US more homogeneous (I^2 = 0 % vs. 87 %) and with a greater effect size than in Europe (SMD 0.37 vs. 0.29, p = 0.03).<br>- Studies including care manager had a greater effect size (SMD 0.42 vs. 0.13, p =0.001)<br>- Studies using stepped collaborative care (2) had a greater effect size than those which didn't (SMD 0.57 vs. 0.29, p =0.04)

Panagioti, 2016	- CC associated with a small but significant effect on depression outcomes at 4 to 6 months follow-up (standardized mean difference [SMD], −0.22 [95% CI, −0.25 to −0.18]; I^2 = 0.8%; 0.3%-3.5%)<br>- Individual participant data analyses found no significant interaction effects, indicating that the presence (interaction coefficient, 0.02 [95% CI, −0.10 to 0.13]), numbers (interaction coefficient, 0.01 [95% CI, −0.01 to 0.02]), and types of chronic physical conditions do not influence the treatment effect
Sighinolfi, 2014	- CC more effective than treatment as usual in improving depression outcomes (focus on European countries)<br>- Higher efficacy for the CC interventions delivered in the high fidelity subgroup (studies that completely fulfilled all Gunn et al.'s CC criteria); CC in the low fidelity subgroup didn't seem more effective than the usual care (at short, medium and medium–long term follow-up)
Thota, 2012	- Meta-analyses: CC improved depression symptoms (SMD= 0.34); adherence to treatment (OR=2.22); response to treatment (OR=1.78); remission of symptoms (OR=1.74); recovery from symptoms (OR=1.75); quality of life/functional status (SMD=0.12); and satisfaction with care (SMD=0.39) for patients diagnosed with depression (all effect estimates significant).<br>- Interventions implemented by community-based organizations demonstrated the largest effects, and those in Veterans Administration Centers the smallest.<br>- Type of case manager: effect estimates were largest for nurses and smallest for master's-level mental health workers<br>- Smaller effect estimate for interventions that included "support for self-care" as an element<br>- Negative relationship between number of collaborative care elements and depression symptom improvement (slope= -0.09 SDs/element, p=0.0006)
van Steenbergen-Weijenburg, 2010	- CC for the treatment of depressive disorder was more effective than care as usual in terms of depression-free days and QALYs
**Intersectoral collaboration (nursing home, public health)**	
Davies, 2011	- Although there were some improvements in outcomes, the majority of studies showed that the intervention had either mixed or no effect when compared with the control group
Martin-Misener, 2012	- Improvements in chronic disease management including screening and self-care; improvements in communicable disease control and immunization rates; improvements were seen in maternal and child health (quantitative data not given in the review)
[a]CC: Collaborative care; CI: Confidence Interval; DRPs: Drug-related problems; HbA1c: Hemoglobin bA1c; IPC: Interprofessional collaboration; SMD: Standardized mean difference; QALYs: Quality-adjusted life years; QoL: Quality of life	

Table S5: Detailed results of included reviews for healthcare professional, organizational and cost outcomes[a] (n=13)

Author, year	Healthcare professional outcomes	Organizational outcomes	Cost outcomes
**Interprofessional collaboration in primary care (large scope)**			
Barrett, 2007	- HCP more satisfied and have a more positive experience compared to HCP working in uni-professional model<br>- PCP develop a positive perception of working collaboratively with other professionals<br>- PCP develop enhanced knowledge and skills;<br>- PCP and HCP have different practice behaviors (referral patterns, follow-up, preventive care)	- IPC models can provide a broader range of services, more efficient resource utilization, better access to services, shorter wait times, better coordination of care and more comprehensive care	- There are findings of interprofessional collaboration cost benefits in some primary healthcare settings (such as decreased average provider and patient costs for blood pressure control, lower readmission rates and costs for team-managed, home-based PC)
Gougeon, 2017			- Increase of specific costs or use of services<br>- Overall costs (three studies) do not seem to differ significantly by the end of the study
Trivedi, 2013			- Case Management, collaboration and integrated team all showed mixed evidence

Author, year	Healthcare professional outcomes	Organizational outcomes	Cost outcomes
Wranik, 2019	- Addition of nurses or the expansion of their role improved satisfaction of providers<br>- Addition of pharmacists was more challenging, but collaboration with GP proved possible, with limited impact on practice.<br>- Strong evidence that increasing the ratio of non-clinical to clinical staff had a negative impact on team climate		
**Primary care provider – specialty care provider collaboration**			
Carmont, 2017	- GP engagement with secondary services in integrated care at the end of life, case conferences, home conferences and a good practice framework showed improvements in communication, relationships between services and professional development		

**Author, year**	**Healthcare professional outcomes**	**Organizational outcomes**	**Cost outcomes**
Mitchell, 2002	- Improved clinical behavior for GP (4 studies): more rational use of resources and diagnostic tests (by both GP and specialists), improved clinical skills, more frequent use of appropriate treatment strategies (for example, better rates of referral to community services), and more frequent clinical behaviors designed to detect disease complications (for example, more patients owning peak flow meters in asthma and performing fundoscopy in diabetics)		- Comparisons between general practice-based interventions VS standard outpatient care used very different methods of measuring direct costs; meaningful conclusions were impossible<br>- Community care of chronic psychiatric patients: significant reductions in hospital bed days and longer time to readmission (1 study), but another could draw no conclusions because of wide patient variation<br>- Involving GP in multidisciplinary care adds costs related to communications between team and GP; cost of extra GP consultations in cases – costs may be offset by long-term savings<br>- Insufficient data, diversity of settings and methods of analysis did not allow to estimate any relative cost efficiencies to be made
Mitchell, 2015	- Improved clinical GP performance, better recording of important clinical info & capture of diabetes patient on practice diabetes registers<br>- Improved patient sharing info between sectors<br>- Clinicians reported satisfaction with initiatives (with clinicians holding view that intervention improved patient outcomes)		- Reduction of clinic-based care for diabetes patients compared to hospital outpatient-based care (1 study)<br>- Modest extra costs or no difference (3 studies)<br>- Increased cost for intervention (1 study)<br>- RCT design: costs were higher for intervention (2 studies); other designs: costs were lower (1 study) and no difference to controlled data for other studies<br>- Concerns related to cost of clinic model, impact of model on existing services, and uncertainty of future funding<br>- Cost of integrated model reported as equivalent to traditional alternatives<br>- Additional costs balanced out by social gains<br>- Extra costs attributed to more frequent follow-up appointments, higher cost of community-based pathology services, time required for chart audits

Author, year	Healthcare professional outcomes	Organizational outcomes	Cost outcomes
	- GP concerns: inadequate information regarding purpose and function of clinic; longer waiting times and suboptimal communication with specialists;<br>- Referring GP concerns: unnecessary referrals could de-skill GP, were fearful of having no back-up		and patient home visits or more intense care regimen of the community model
Smith, 2017			- Analysis of costs of shared care: limited by varying effects on effectiveness; study findings suggest shared care is cost-effective for depression<br>- Patient direct costs are lower with shared care than with hospital outpatient care, mainly due to reduced travel costs
**Primary care provider – mental health care provider collaboration ("Collaborative care")**			
Butler, 2008			- IMPACT studies: several cost effectiveness calculations suggesting modest added treatment costs in light of benefits; suggested achieved actual net savings, but basis for calculations is unclear<br>- A few other studies show higher costs per QALY but still well below typical thresholds<br>- Anxiety disorder studies with integrated care programs may hold more potential, with improved outcomes for reduced costs.<br>- Integrating Primary Care into Specialty Mental Health (3 studies): two programs found to be cost-neutral (increases in outpatient expenditures offset by declines in inpatient and emergency room use); significant decline in annual costs for a subsample of patients with substance-related mental and medical comorbidities compared to the control group.

**Author, year**	**Healthcare professional outcomes**	**Organizational outcomes**	**Cost outcomes**
Dham, 2017			- overall increased cost with use of CC, but studies suggest a cost benefit when accounting for depression-free days and QALYs<br>- IMPACT study: total outpatient costs were USD $295 higher during the study period, but cost-effective after accounting for QALYs<br>- PRISM-E study: cost benefit in Veterans Affairs settings but telemedicine had higher costs per QALY than other studies on CC (incremental cost of $85,637/QALY)<br>- Cardiac patients: cost of CC was $175.27 higher per participant but was cost-effective because of depression free days and improvement in QALYs (incremental cost-effectiveness of $3,337.06/QALY)
Fuller, 2011 a			- Limited economic data; studies used different measures for costs and benefits, different timeframes and economic indicators<br>- Three of the four studies using linkages across the most common combination of broad linkage category reported positive economic outcomes<br>- Over a half of the economic studies reported costs were lower, the same or acceptably higher given additional clinical and service delivery benefits obtained<br>-IMPACT: average cost per patient of the intervention was US$591, incremental outpatient cost per depression-free day US$2.76, and cost per QALY was $2,519; thought similar to other mainstream treatments.<br>- PRISM-E: no economic effectiveness reported.

**Author, year**	**Healthcare professional outcomes**	**Organizational outcomes**	**Cost outcomes**
van Steenbergen-Weijenburg, 2010			- increment in intervention related direct medical costs of $519 (1 study), $675 (1 study) and $921 for a two-year follow-up, compared with CAU (1 study)<br>- Costs over 2 years of $21,148 (SD $27,548) for the intervention group vs. $22,258 (SD $35,607) for the control group, a saving of $1,110 (1 study)<br>- Increment of $1,974 for outpatient costs (contacts with medical or ancillary providers and medications) and inpatient health services costs (hospitalization, inpatient services and procedures, medications) together<br>- Direct non-medical costs: time for outpatient medical (30 minutes) and mental health visits (45 minutes) (1 study); travelling expenses from $1,636 (95% CI $1,356-$1,916) for intervention group to $1,337 (95% CI $1,174-$1,499) for CAU group (1 study)<br>- Indirect non-medical costs: significant intervention effect on days worked overall, but no statistical difference in amount of sick days between intervention and usual care patients<br>- CC intervention for MDD can be cost-effective, relative to usual care, depending on willingness to pay<br>- CC is effective but in most cases is also more expensive than CAU<br>- Cost-utility: direct medical intervention costs per QALY varied from $2519 (95% CI -4,517 - $9,554) to $5037 (95% CI -$9,034 to $19,108) (1 study); direct- and indirect costs per QALY together were $21,478 (1 study); costs per QALY at $49,500 (1 study).<br>- CC for depressive disorder: incremental costs per QALY were $21,478 to $49,500 for all the costs of healthcare services together.
**Intersectoral collaboration**			

Author, year	Healthcare professional outcomes	Organizational outcomes	Cost outcomes
Martin-Misener, 2012	- Enhanced educational experiences for students and development of new academic programs<br>- Improvements in understanding of PC and PH concepts, areas of responsibility and roles, team functioning and information sharing (practice level).	- Improved access to care and quality of care.<br>- Improved efficiencies through timelier case reporting and less duplication of care<br>- Improved continuity and coordination of care<br>- Care delivery processes strengthened	- Increased funding support and enhanced sustainability (from IPC and resource sharing).
[a]CAU: Care as Usual; CC: Collaborative Care; MDD: Major Depressive Disorder; PC: Primary Care; PH: Public Health; QALYs: Quality-Adjusted Life-Years			
